# B cell senescence promotes age‐related changes in oral microbiota

**DOI:** 10.1111/acel.14304

**Published:** 2024-08-09

**Authors:** Hiroya Mizuno, Shimpei Kawamoto, Ken Uemura, Jeong Hoon Park, Nozomi Hori, Yumiko Okumura, Yusuke Konishi, Eiji Hara

**Affiliations:** ^1^ Department of Molecular Biology, Research Institute for Microbial Diseases Osaka University Suita Osaka Japan; ^2^ Laboratory of Aging Biology, Immunology Frontier Research Center Osaka University Suita Osaka Japan; ^3^ Center for Infectious Disease Education and Research Osaka University Suita Osaka Japan

**Keywords:** B cell, cellular senescence, immunoglobulin A, microbiota, saliva

## Abstract

In recent years, there has been increasing attention towards understanding the relationship between age‐related alterations in the oral microbiota and age‐associated diseases, with reports emphasizing the significance of maintaining a balanced oral microbiota for host health. However, the precise mechanisms underlying age‐related changes in the oral microbiota remain elusive. We recently reported that cellular senescence of ileal germinal center (GC) B cells, triggered by the persistent presence of commensal bacteria, results in diminished IgA production with aging and subsequent alterations in the gut microbiota. Consequently, we hypothesize that a similar phenomenon may occur in the oral cavity, potentially contributing to age‐related changes in the oral microbiota. Examination of *p16‐luc* mice, wherein the expression of the senescent cell marker p16^INK4a^ can be visualized, raised under specific pathogen‐free (SPF) or germ‐free (GF) conditions, indicated that, unlike ileal GC B cells, the accumulation of senescent cells in GC B cells of cervical lymph nodes increases with age regardless of the presence of commensal bacteria. Furthermore, longitudinal studies utilizing the same individual mice throughout their lifespan revealed concurrent age‐related alterations in the composition of the oral microbiota and a decline in salivary IgA secretion. Further investigation involving *Rag1* ^*−/−*^ mice transplanted with B cells from wild‐type or *p16* ^*INK4a*^ and *p21* ^*Waf1/Cip1*^ ‐double knockout mice unveiled that B cell senescence leads to reduced IgA secretion and alteration of the oral microbiota. These findings advance our understanding of the mechanism of age‐associated changes in the oral microbiota and open up possibilities of their control.

## Graphical abstract

At a young age, GC B cells induced by oral antigens (bacteria, viruses, etc.) contribute to the secretion of sufficient salivary IgA into the oral cavity, leading to the symbiotic relationship between the host and oral microbiota (left). However, the induction of cellular senescence in the GC B cells by long‐term stimulation of oral antigens with aging leads to a decrease in IgA secretion in saliva, causing age‐related changes in the oral microbiota, which may result in dysbiosis (right).

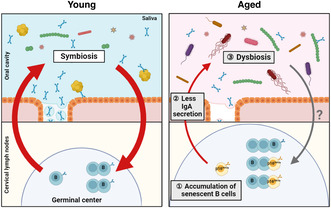

**Abbreviations**
ABC: age‐associated B cells
Aicda: activation‐induced cytidine deaminase
ANOVA: analysis of variance
ASV: amplicon sequence variant
Bcl6: B cell leukemia/lymphoma 6
BLI: bioluminescence imaging
Bmi1: Bmi1 polycomb ring finger oncogene
Cdkn1a: cyclin dependent kinase inhibitor 1a
Cdkn2a: cyclin dependent kinase inhibitor 2a
CRC: colorectal cancer
DKO: double knockout
EdU: 5‐ethynyl‐2'‐deoxyuridine
ELISA: enzyme‐linked immunosorbent assay
FACS: fluorescence‐activated cell sorting
Fas: Fas cell surface death receptor
GC: germinal center
GF: germ‐free
IgA: immunoglobulin A
MaAsLin: Microbiome Multivariable Association with Linear Models
MMTV: mouse mammary tumor virus
PCoA: principal coordinate analysis
PNA: peanut agglutinin
Rag1: recombination activating 1
rRNA: ribosomal RNA
RT‐qPCR: reverse transcription‐quantitative polymerase chain reaction
S1pr2: sphingosine‐1‐phosphate receptor 2
SARS‐CoV‐2: severe acute respiratory syndrome coronavirus 2
SASP: senescence‐associated secretory phenotype
SAT: senescence‐associated T cells
scRNAseq: single‐cell RNA sequencing
SPF: specific pathogen‐free
UMAP: uniform manifold approximation and projection
WT: wild type
γH2A.X: phosphorylated histone variant H2A.X

## 1 BACKGROUND

In recent years, the role of commensal microbiota inhabiting various anatomical sites such as the oral cavity, nasal mucosa, and the intestinal tract has garnered significant attention due to its profound effects on host physiology (J. L. Baker et al., 2024; de Vos et al., 2022; Konkel et al., 2019). Among these, the gut microbiota, being the largest and most studied, is crucial for maintaining host health, with dysbiosis (a state characterized by an imbalance of microbiota) implicated in the pathogenesis of numerous diseases (Konkel et al., 2019; Kostic et al., 2013; Levy et al., 2017; Roy et al., 2017). Even more noteworthy is the possibility that changes in the oral microbiota may influence abnormalities in the gut microbiota. For example, it is increasingly recognized that ectopic growth of oral bacteria in the intestinal tract plays an important role in the pathogenesis of many diseases, including inflammatory bowel diseases and colorectal cancer (CRC) (Atarashi et al., 2017; Kitamoto et al., 2020; Pacheco‐Yanes et al., 2023). Indeed, the oral bacteria that cause periodontal diseases, such as *Fusobacterium nucleatum*, are reportedly increased in the intestinal tract of CRC patients and contribute to its development (Okumura et al., 2021; Wang & Fang, 2023; Willis & Gabaldón, 2020). Furthermore, several lines of evidence indicate that an increase in oral bacteria in the intestinal tract may contribute to age‐related dysbiosis of gut microbiota (Iwauchi et al., 2019; Odamaki et al., 2016). Considering that abnormalities in the oral microbiota have also been reported to occur with aging (Willis et al., 2022), the increase in oral bacteria in the intestinal tract with aging may be due to the transfer of abnormally increased oral bacteria into the intestinal tract through saliva swallowing. However, the precise mechanisms underlying age‐related dysbiosis of the oral microbiota remain unclear.

Among various host‐derived factors known to be involved in the regulation of commensal microbiota, immunoglobulin A (IgA), which is abundantly secreted on mucosal surfaces, is thought to play an important role (Chen et al., 2020). IgA is known to regulate the balance of the commensal microbiota by binding to bacteria and contributing to the promotion or elimination of bacterial colony formation, depending on the type of bacteria (Donaldson et al., 2018; Kawamoto et al., 2014; Macpherson et al., 2018; Palm et al., 2014). Notably, dysbiosis of the gut microbiota stemming from IgA deficiency has been observed in both human and murine models (Catanzaro et al., 2019; Fadlallah et al., 2018; Fagarasan et al., 2002; Moll et al., 2021; Suzuki et al., 2004), with implications for the development of autoimmune disorders through aberrant immune activation (Kawamoto et al., 2012; Nagaishi et al., 2022). IgA is secreted not only in the intestinal tract but also in other mucosal sites such as the oral cavity. While there is already evidence suggesting the involvement of IgA in the regulation of oral microbiota (Berbers et al., 2020; Scholz et al., 2014), the impact of IgA on age‐related changes in oral microbiota and its mechanisms remain unclear.

Cellular senescence is a state of irreversible cell cycle arrest induced by various stresses such as telomere dysfunction, oncogene activation, radiation, ultraviolet light, DNA‐damaging agents, and oxidative stress. Since many of these stresses have the potential to increase the risk of tumor formation, cellular senescence is considered to function as a crucial tumor suppressor mechanism (Gorgoulis et al., 2019). However, cellular senescence in stem or progenitor cells reduces the stem cell pool and impairs tissue homeostasis (Di Micco et al., 2021). Furthermore, senescent cells also cause senescence‐associated secretory phenotypes (SASP), in which the cells secrete a variety of pro‐inflammatory factors into the extracellular fluid (Acosta et al., 2008; Chaib et al., 2022; Coppé et al., 2008; Kuilman et al., 2008; Wu et al., 2024). Therefore, the accumulation of senescence‐like cells, which is often seen with aging and/or obesity (Krishnamurthy et al., 2004; López‐Otín et al., 2023; Yamakoshi et al., 2009; Yoshimoto et al., 2013), ultimately leads to harmful side effects (D. J. Baker et al., 2016; Chan & Narita, 2019; Mehdizadeh et al., 2022; Ogrodnik et al., 2017). Moreover, our recent studies have shown that age‐associated cellular senescence of ileal germinal center (GC) B cells induced by commensal bacteria reduces IgA production and diversity, leading to gut dysbiosis (Kawamoto et al., 2023). Based on these findings, we considered the possibility that similar mechanisms may operate in the oral immune system, contributing to abnormalities in the oral microbiota with aging.

## 2 RESULTS

### 2.1 Accumulation of senescent cells in cervical lymph nodes regardless of the presence of commensal bacteria

**FIGURE 1:**
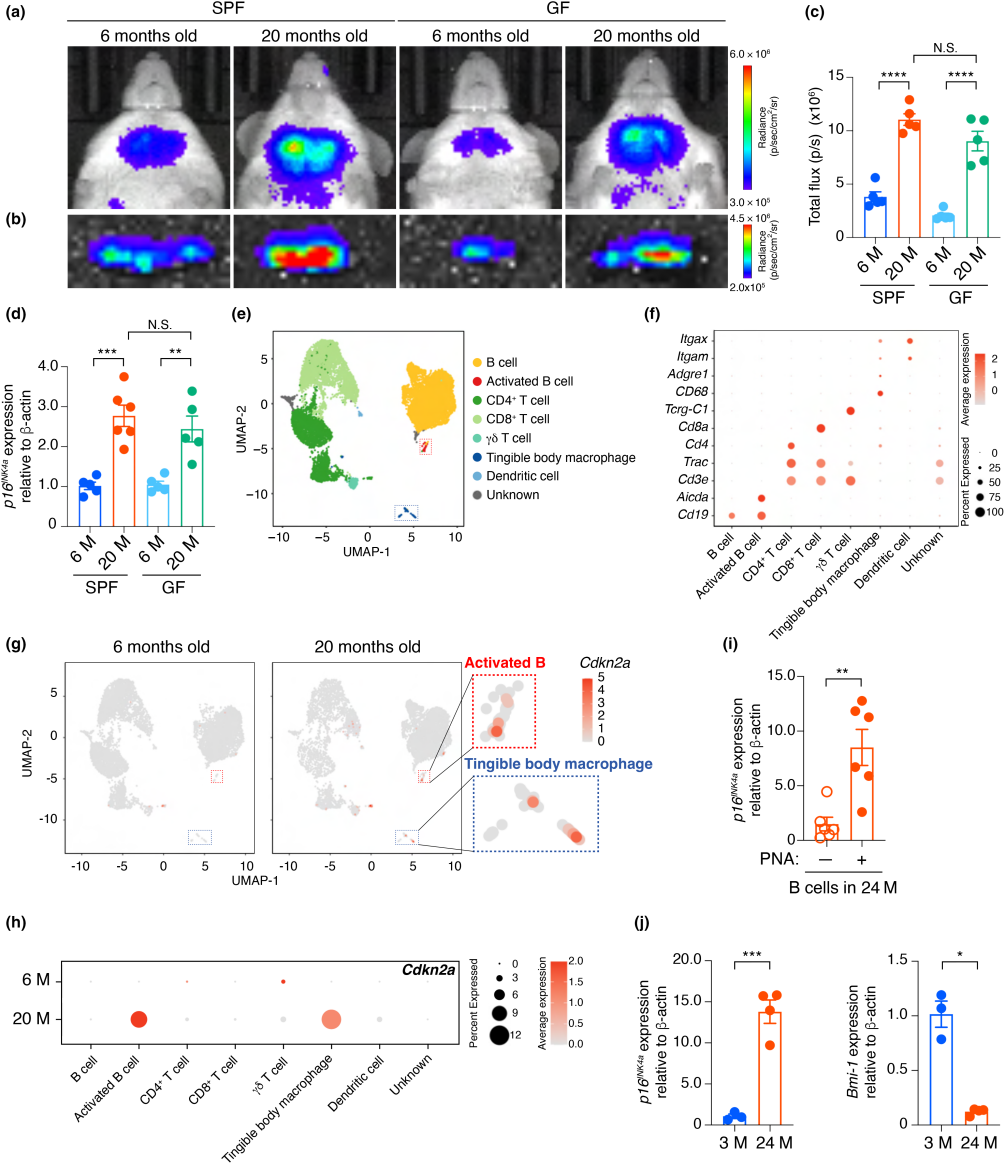
Bacteria‐independent induction of *p16* ^*INK4a*^ expression in cervical lymph nodes with aging. (a) and (b), Representative images of noninvasive BLI (a) or ex vivo BLI of cervical lymph nodes (b) of *p16‐luc* mice (male) raised in SPF or GF environment. The color bars indicate the radiance with minimum and maximum threshold values. (c) The bioluminescence intensity emitted from the cervical lymph nodes. (d) The cervical lymph nodes isolated from SPF and GF mice (male) were subjected to analysis of RT‐qPCR for *p16* ^*INK4a*^ expression. (e–h) CD45^+^ cells isolated from the cervical lymph nodes of 6‐ or 20‐month‐old SPF mice (male) were subjected to scRNA‐seq analysis. UMAP plot gathers all CD45^+^ cells from cervical lymph nodes from 6 or 20 M SPF WT mice, in which all cells are clustered and color‐coded by cell types (e). Dot plot showing the marker genes used for the cluster classification (f). UMAP plots of cells expressing *Cdkn2a* are shown as red dots (g). Note that *Cdkn2a* expressing cells are enriched in the cluster of activated B cells and tingible body macrophage, which are enclosed by the red and blue dotted line, respectively (g, h). (i) RT‐qPCR analysis of *p16* ^*INK4a*^ expression in PNA^−^ or PNA^+^ (GC) B cells isolated from cervical lymph nodes of 24‐month‐old WT SPF mice (male). (j) RT‐qPCR analysis of *p16* ^*INK4a*^ (left) or *Bmi‐1* expression (right) in B cells isolated from cervical lymph nodes of 3‐ or 24‐month‐old WT mice (male). Sample sizes (n) indicate the number of biologically independent animals (*n* = 5 or 6 in (c, d), *n* = 6 in (i) *n* = 3 or 4 in (j)). The data are indicated as means ± s.e.m. Statistical significance was determined with two‐way ANOVA followed by Šídák's multiple comparisons test (c, d) and two‐tailed unpaired *t* test (i, j). M, months; N.S., not significant. *p < 0.05, **p < 0.01, ***p < 0.005, ****p < 0.001.

To investigate the relationship between commensal bacteria and cellular senescence in cervical lymph nodes during aging, we took advantage of the *p16* ^*INK4a*^‐reporter mice (*p16‐luc* mice), in which the dynamics of the expression of *p16* ^*INK4a*^, a senescence‐inducing gene (Hara et al., 1996) can be monitored throughout the body using a bioluminescence imaging (BLI) technique (Yamakoshi et al., 2009). In vivo BLI was performed in specific pathogen‐free (SPF) and germ‐free (GF) environments over a lifetime. As we previously reported (Yamakoshi et al., 2009), the bioluminescence signal in the neck increased substantially with age in *p16‐luc* mice in the SPF environment (Figure 1a). Notably, this was also the case in the GF environment (Figure 1a), and ex vivo imaging of the excised cervical lymph nodes revealed that the increased bioluminescence in the neck was mainly attributed to cervical lymph nodes (Figure 1b,c). Furthermore, RT‐qPCR analysis confirmed that the expression level of endogenous *p16* ^*INK4a*^ in cervical lymph nodes increases with age in both SPF and GF mice (Figure 1d). Thus, unlike in the ileum (Kawamoto et al., 2023), *p16* ^*INK4a*^‐expressing cells in cervical lymph nodes accumulate with age independently of commensal bacteria.

### 2.2 Aging leads to the accumulation of senescent germinal center B cells in cervical lymph nodes

To identify the cells undergoing cellular senescence in cervical lymph nodes, we next performed single‐cell RNA sequencing (scRNAseq) analysis using cells isolated from cervical lymph nodes of 6‐ or 20‐month‐old SPF mice (Figure 1e,f). The scRNAseq analysis revealed that the expression level of the *Cdkn2a*, which encodes both the *p16* ^*INK4a*^ and *p19* ^*ARF*^genes (Gil & Peters, 2006), is markedly increased in the population of activated B cells and tingible body macrophages with aging (Figure 1e–h). Furthermore, the activated B cell population expressing the *Cdkn2a* also expresses other senescence‐inducing gene, such as *Cdkn1a* (*p21* ^*Waf1/Cip1*^), as well as a series of genes known to be expressed in GC B cells, such as *Aicda*, *Bcl6*, *Fas*, and *S1pr2* (Figure S1a,b). RT‐qPCR confirmed that GC B cells isolated from cervical lymph nodes of aged mice that were PNA positive had significantly increased expression of *p16* ^*INK4a*^ (Figure 1i). Furthermore, the age‐related upregulation of *p16* ^*INK4a*^ is inversely correlated with the level of *Bmi‐1*, a negative regulator of the *p16* ^*INK4a*^ gene (Jacobs et al., 1999), suggesting that GC B cells in cervical lymph nodes likely undergo cellular senescence with age (Figure 1j).

**FIGURE 2:**
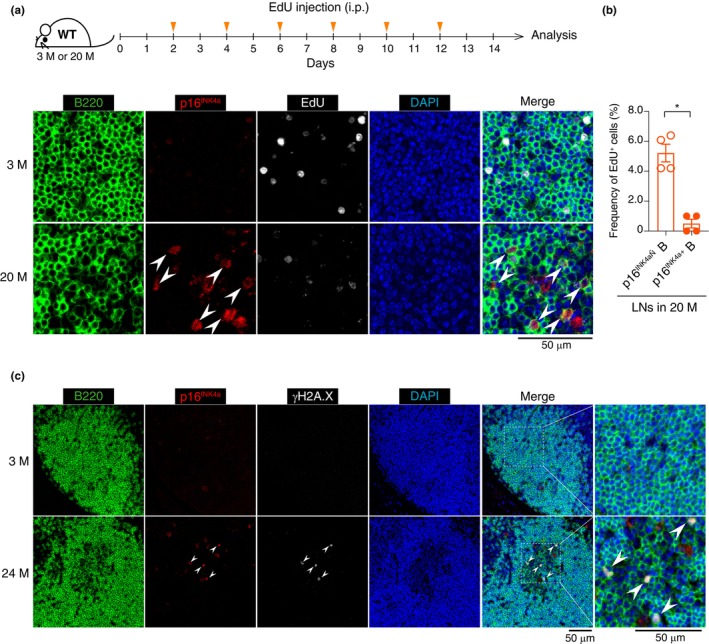
Induction of cellular senescence in GC B cells in cervical lymph nodes with aging. (a) Timeline of the experimental procedure of EdU injection using 3‐ or 20‐month‐old WT mice (top). Immunohistochemical images of cervical lymph nodes stained with B220, p16^INK4a^, and EdU from 3‐ or 20‐month‐old WT SPF mice injected with EdU (bottom). Scale bar, 50 μm. (b) Frequency of EdU^+^ cells in p16^INK4a^‐negative or ‐positive B cells in cervical lymph nodes of 20‐month‐old WT mice. (c) Immunohistochemical images of cervical lymph nodes stained with B220, p16^INK4a^, and γH2A.X from 3‐ or 24‐month‐old WT SPF mice (male). Scale bar, 50 μm. Sample sizes (n) indicate the number of biologically independent animals (*n* = 4 in (b)). The data are indicated as means ± s.e.m. Statistical significance was determined with Mann–Whitney *U* test (b). i.p., intraperitoneal. *p < 0.05, **p < 0.01, ***p < 0.005, ****p < 0.001.

To further explore this possibility, we next performed EdU (5‐ethynyl‐2′‐deoxyuridine) uptake analysis in combination with immunostaining for endogenous p16^INK4a^ and B220, a B cell marker, in 3‐month‐old and 20‐month‐old SPF mice (Figure 2a). We observed that GC B cells expressing p16^INK4a^ in aged cervical lymph nodes had substantially lower uptake of EdU than cells not expressing p16^INK4a^ (Figure 2a,b). Note that p16^INK4a^ was detected in 15‐month‐old cervical lymph nodes of wild‐type (WT) mice, but not in *p16* ^*INK4a*^/*p21* ^*Waf1/Cip1*^‐double‐knockout mice (Figure S2). Furthermore, the p16^INK4a^ signal coincided with γH2A.X foci, a sign of the DNA damage response associated with cellular senescence (Figure 2c), indicating that GC B cells in cervical lymph nodes undergo cellular senescence in aged mice.

### 2.3 Age‐related changes in oral microbiota

**FIGURE 3:**
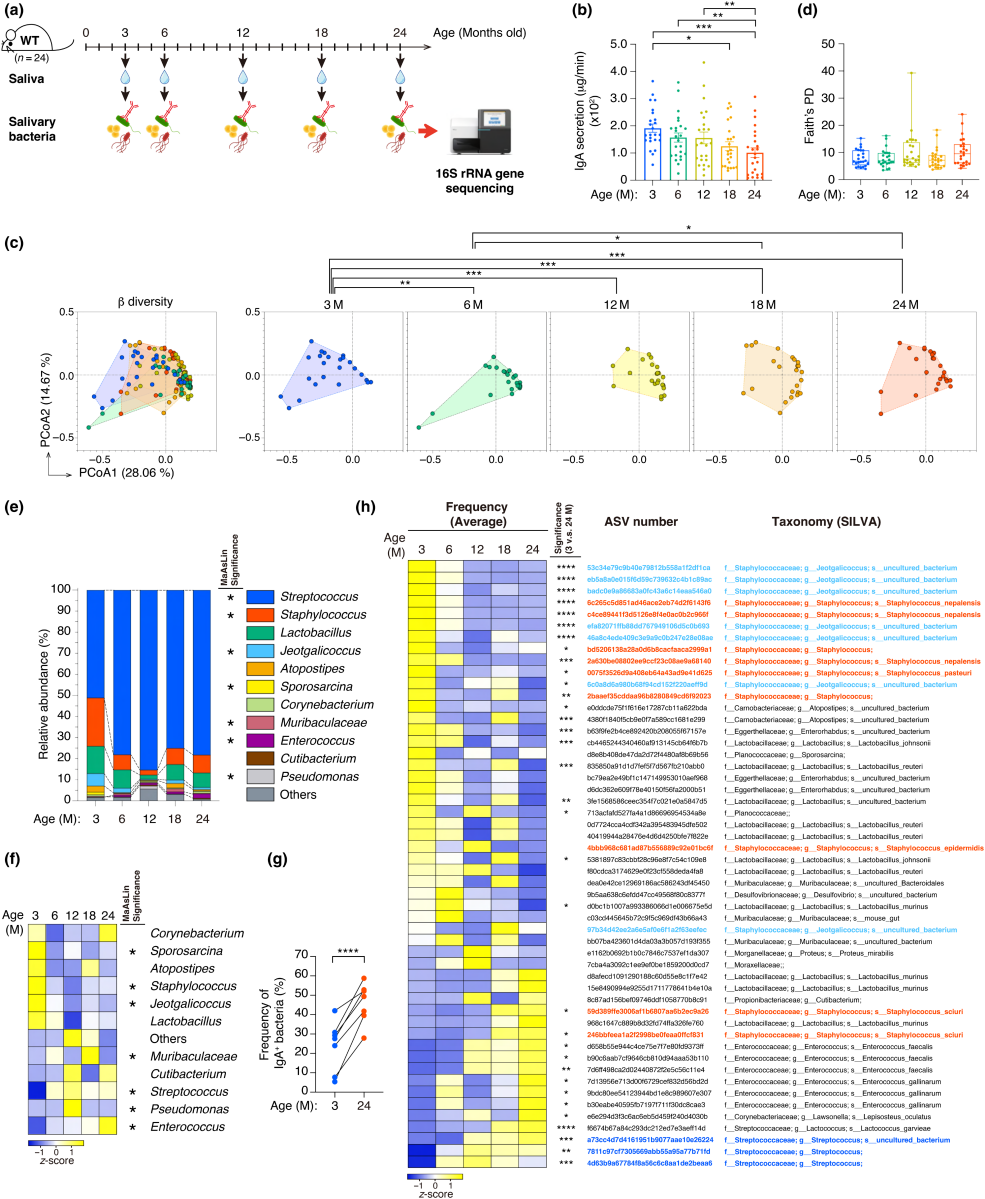
Age‐related changes in oral microbiota and salivary IgA. (a) Timeline of follow‐up experiments to analyze age‐related changes in the oral microbiota and IgA (*n* = 24). (b) IgA secretion in the saliva collected at each month old (3, 6, 12, 18, and 24 months old) of the same individual WT mice was determined by ELISA. (c) Principal Coordinate Analysis (PCoA) plots of the Bray‐Curtis distance indicate changes in oral bacterial composition with aging (3, 6, 12, 18 and 24 M). PCoA plots for each age group are indicated on the right. The statistical significance judged by PERMANOVA is indicated at the top. (d) Alpha diversity (Faith's PD) of oral microbiota at each month old (3, 6, 12, 18, and 24 M). There were no statistically significant differences between any groups as judged by Kruskal–Wallis test. (e) Bar plots indicating the phylogenetic composition of the oral microbiota of mice at different ages at the genus level. The bacterial genera with statistically significant differences judged by MaAsLin are marked with an asterisk. (f) Heat map showing age‐related changes of bacterial genera. The degree of blue or yellow color intensity means a negative or positive *z* score, respectively (f, leftmost column). The bacterial genera with statistically significant differences judged by MaAsLin are marked with an asterisk (f, middle column). (g) Frequency of IgA^+^ bacteria in the saliva collected at 3 and 24 months old of the same individual WT mice. (h) Age‐related changes in the abundance of specific bacterial species (ASVs) in saliva over time in 24 mice. The heat map shows age‐related changes in bacteria with statistically significant differences judged by MaAsLin. The degree of blue‐ or yellow‐color intensity indicates a negative or positive z‐score, respectively (h, left column). The ASVs showing a statistically significant difference between the bacterial frequency in 3‐ and 24‐month‐old mice are marked with an asterisk (*n* = 24) (h, second‐left column). The ASV number and name of the bacterial taxonomy classified by SILVA are represented on the right. ASVs classified as *Staphylococcus*, *Streptococcus*, or *Jeotgalicoccus* were highlighted by red, blue, or cyan, respectively. Sample sizes (n) indicate the number of biologically independent animals (*n* = 24 in (a‐f) and (h), and *n* = 7 in (g)). The data are indicated as means ± s.e.m. (b). Statistical significance was determined with Friedman rank‐sum test followed by pairwise Wilcoxon signed rank test (b), two‐tailed paired *t* test (h), or Wilcoxon matched‐paired signed rank test (g). *p < 0.05, **p < 0.01, ***p < 0.005, ****p < 0.001.

Given the pivotal involvement of GC B cells in the production of IgA, a key regulator of the commensal microbiota, we hypothesize that cellular senescence of GC B cells may promote a decrease in IgA secretion in the oral cavity, resulting in age‐related changes in the oral microbiota. The commensal bacteria of mice is known to be greatly influenced by various factors such as parentage, rearing cage, and sex (Beale et al., 2019; G. Singh et al., 2021). Therefore, it is expected that there will be some degree of variation in the oral microbiota among individuals. Previous studies have investigated how the oral microbiota changes with age in both mice and humans (Horev et al., 2020; Willis et al., 2022). However, these studies compared the oral microbiota between different individuals, which leaves the possibility that the observed changes could be due to differences between individuals rather than age‐related changes. Therefore, in the present study, we decided to follow the changes in IgA levels and oral microbiota with aging over the lifetime of the same individual mice. Saliva samples were collected from 24 WT mice housed in different cages at 3, 6, 12, 18 and 24 months of age to examine age‐related changes in IgA level and oral microbiota (Figure 3a). Notably, along with a decrease in salivary IgA secretion (Figure 3b), there were significant age‐related compositional changes in the salivary microbiota, as judged by Principal Coordinate Analysis (PCoA) using meta‐16S rRNA gene sequence dataset (Figure 3c). However, no significant differences were observed in alpha diversity across all groups (Figure 3d). Next, in order to identify bacterial genera exhibiting significant changes with aging, we performed MaAsLin analysis, a cross‐nested abundance analysis that takes into account factors influencing the microbiota dynamics, such as parental difference, housing conditions, and sex disparities (Mallick et al., 2021). Interestingly, among the 11 major bacterial genera of the oral microbiota, *Streptococcus* was identified as increasing with age, whereas *Staphylococcus* and *Jeotgalicoccus* were identified as decreasing, although this trend was more pronounced at 12 months of age than at 18 and 24 months of age (Figure 3e,f). Note that the secretion of salivary IgA decreased significantly with age (Figure 3b), while the frequency of IgA‐binding bacteria in saliva showed a marked increase with age (Figure 3g), suggesting that not only the production of IgA but also the binding affinity or specificity of IgA to oral bacteria may change with age.

Next, we performed a similar analysis at the bacterial species level (ASV) to identify the bacteria that significantly change with aging among these genera. We identified the following bacterial species as significantly changing bacteria with age: three unspecified species of *Streptococcus*, seven unspecified species of *Jeotgalicoccus*, and *Staphylococcus nepalensis*, *Staphylococcus pasteuri*, *Staphylococcus sciuri*, *Staphylococcus epidermidis* and two unspecified species of *Staphylococcus* (Figure 3h and Figure S3). Among the three ASVs identified as *S. nepalensis* by SILVA, two ASVs (6c265c5d851ad46ace2eb74d2f6143f6 and c4ce89441f3d5126e8f4e0ac0b2c966f) showed high relative abundance. Interestingly, these two ASVs identified as *S. nepalensis* accounted for 10% of the oral microbiota composition at young age (3 M) and declined significantly with aging (Figure 3h and Figure S3). In contrast, among the *Staphylococcus* genus, *S. sciuri* increased with aging, especially from 12 to 24 months old (Figure S3). The slight increase in the *Staphylococcus* genus at 18 months old may be due to the rise of several *Staphylococcus* species, such as *S. sciuri* (Figure S3). Since IgA is known as one of the key factors to regulate the commensal microbiota (Donaldson et al., 2018; Kawamoto et al., 2014; Macpherson et al., 2018; Palm et al., 2014), we evaluated the correlation between IgA secretion and relative abundance of genus or species of bacteria. Some genera and species were significantly correlated with IgA secretion (Tables S4, S5). Interestingly, the abundance of *S. nepalensis* decreased significantly with age, and was positively correlated with IgA secretion (Table S5), suggesting that IgA affects the colonization of *S. nepalensis* in the oral cavity. Thus, we demonstrated that some major bacterial species in oral microbiota change significantly with aging depending on IgA secretion.

### 2.4 The impact of B cell senescence on IgA production and the oral microbiota

**FIGURE 4:**
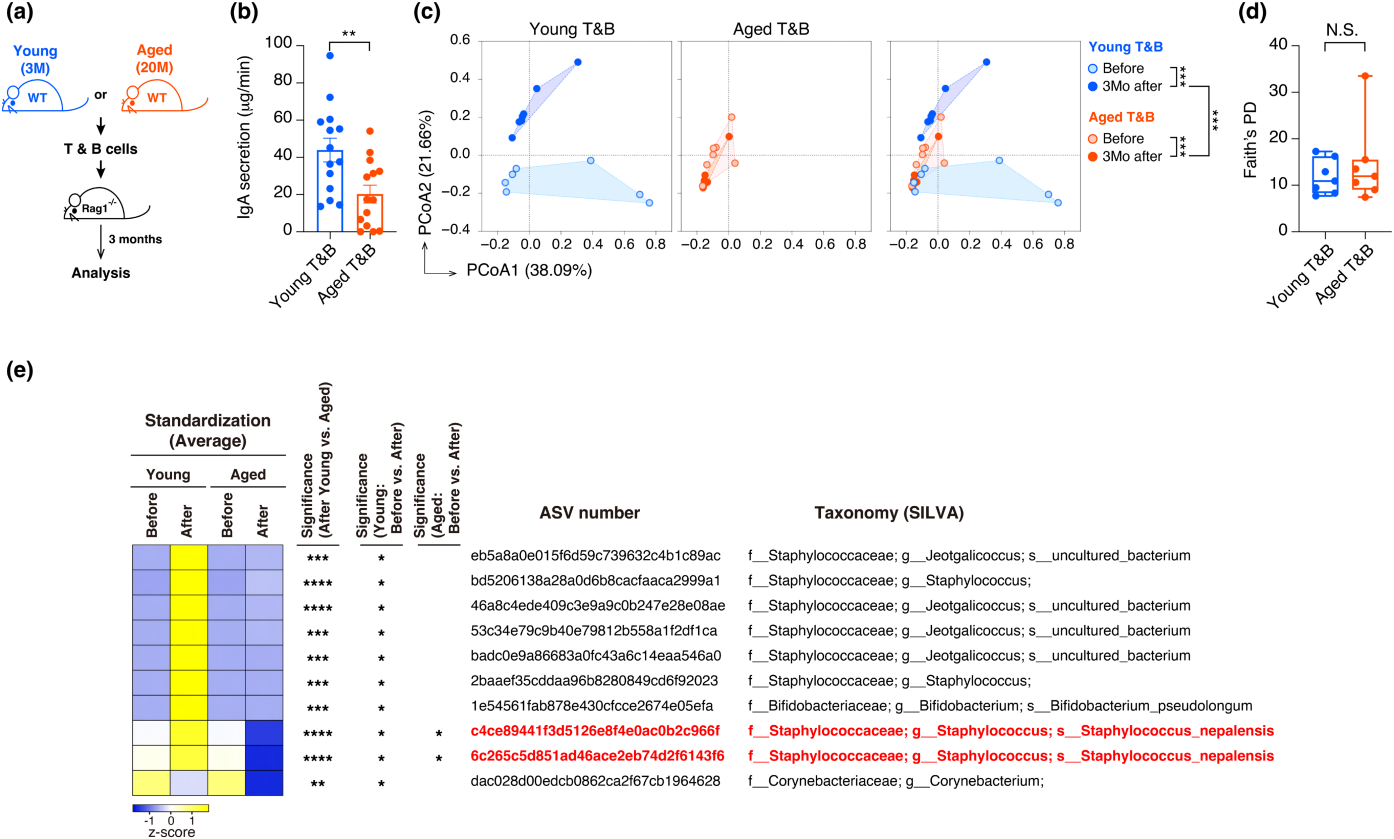
Age‐related immune changes cause the alteration of oral microbiota by reducing salivary IgA secretion. (a) T and B cells isolated from young (3 M) or aged (20 M) WT mice (female) were transplanted into *Rag1* ^*−/−*^ mice (female) and analyzed 3 months after transplantation. Experimental schemes for T‐ and B‐cell reconstitution models using *Rag1* ^*−/−*^ mice are shown. (b) Transplanted mice were examined for IgA secretion in saliva. (c) Comparison of oral microbiota composition before and after T and B cell reconstitution. Principal Coordinate Analysis (PCoA) plots of the Bray‐Curtis distance show changes in oral bacterial composition before and after T and B cell reconstitution. The statistical significance judged by PERMANOVA is shown on the right‐hand side. (d) Alpha diversity (Faith's PD) of oral microbiota in transplanted mice. There were no statistically significant differences between any groups as judged by the Kruskal–Wallis test. (e) Transplantation‐related changes in bacterial species (ASVs) in the saliva of the transferred mice. The standardization values of ASVs were calculated by subtracting the relative abundances of ASVs before and after transplantation. The bacteria that significantly changed in the transferred mice depending on functional IgA were identified as described in materials and methods. The heat map shows the changes in the identified bacteria before and after the transplantation. Negative or positive *z* scores are indicated as the degree of blue or yellow color intensity, respectively (leftmost column). The ASVs with a statistically significant difference in the indicated comparison are marked with an asterisk (second‐, third‐, and fourth‐left columns). The ASV number and name of the bacterial taxonomy classified by SILVA are represented on the right. Sample sizes (n) indicate the number of biologically independent animals (*n* = 14 in (b), *n* = 7 in (c–e)). The data are indicated as means ± s.e.m. Statistical significance was determined with two‐tailed unpaired *t* test (b), Wilcoxon matched‐paired signed rank test (between the relative abundance of “Young‐Before” and “Young‐After”) (between the relative abundance of “Aged‐Before” and “Aged‐After”) (e), Wilcoxon rank‐sum test (between the relative abundance of “Young‐After” and “Aged‐After”) (e). *p < 0.05, **p < 0.01, ***p < 0.005, ****p < 0.001.

**FIGURE 5:**
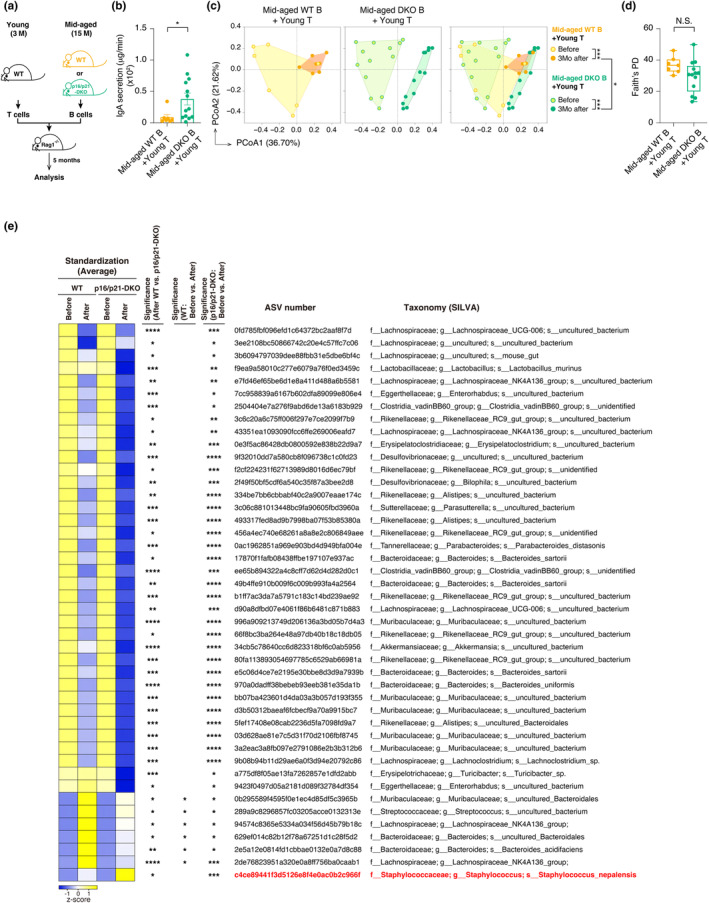
Age‐related induction of senescent B cells causes the alteration of oral microbiota by reducing salivary IgA secretion. (a) T cells isolated from young (3 M) WT mice (female) together with B cells isolated from mid‐aged (15 M) WT or p16/p21‐DKO mice (female) were transplanted into *Rag1* ^*−/−*^ mice (female) and analyzed 5 months after transplantation. Experimental schemes for T‐ and B‐cell reconstitution models using *Rag1* ^*−/−*^ mice are shown. (b) Transplanted mice were examined for IgA secretion in saliva. (c) Comparison of oral microbiota composition before and after T and B cell reconstitution. Principal Coordinate Analysis (PCoA) plots of the Bray‐Curtis distance show changes in oral bacterial composition before and after T and B cell reconstitution. The statistical significance judged by PERMANOVA is shown on the right‐hand side. (d) Alpha diversity (Faith's PD) of oral microbiota in transplanted mice. There were no statistically significant differences between any groups as judged by the Kruskal–Wallis test. (e) Transplantation‐related changes in bacterial species (ASVs) in the saliva of the transferred mice. The standardization values of ASVs were calculated by subtracting the relative abundances of ASVs before and after transplantation. The bacteria that significantly changed in the transferred mice depending on functional IgA were identified as described in materials and methods. The heat map shows the changes in the identified bacteria before and after the transplantation. Negative or positive *z* scores are indicated as the degree of blue or yellow color intensity, respectively (e, leftmost column). The ASVs with a statistically significant difference in the indicated comparison are marked with an asterisk (second‐, third‐, and fourth‐left columns). The ASV number and name of the bacterial taxonomy classified by SILVA are represented on the right. Sample sizes (n) indicate the number of biologically independent animals (*n* = 7 or 13 in (b–e)). The data are indicated as means ± s.e.m. Statistical significance was determined with two‐tailed unpaired *t* test (b), Wilcoxon matched‐paired signed rank test (between the relative abundance of “WT‐Before” and “WT‐After”) (between the relative abundance of “p16/p21‐DKO‐Before” and “p16/p21‐DKO‐After”) (e), Wilcoxon rank‐sum test (between the relative abundance of “WT‐After” and “p16/p21‐DKO‐After”) (e). *p < 0.05, **p < 0.01, ***p < 0.005, ****p < 0.001.

To investigate the effects of age‐related changes in B and T cells on IgA production and the oral microbiota composition, a mixture of T and B cells from young (3 months old) or aged (20 months old) WT mice was transplanted into *Rag1* ^*−/−*^ mice lacking T cells and B cells (Figure 4a). Notably, *Rag1* ^*−/−*^ mice transplanted with T and B cells from young mice exhibited a marked increase in IgA secretion compared to mice transplanted with T and B cells from aged mice (Figure 4b). Additionally, a dramatic change in the composition of the oral microbiota was observed after transplantation (Figure 4c), although there were no significant differences in alpha diversity between the two groups (Figure 4d). To investigate the impact of IgA on the reconstruction of the oral microbiota, we attempted to identify oral bacteria that are significantly changed after the transplantation of T and B cells isolated from young mice. Interestingly, *S. nepalensis*, which was significantly decreased with age (Figure 3h and Figure S3), was found to be markedly increased after the complete reconstitution of IgA in the mice transferred with young T and B cells, suggesting that IgA regulates colonization of *S. nepalensis* in the oral cavity (Figure 4e). These results indicate that T and B cells from aged mice have a reduced ability to produce IgA, which is required for the establishment of the oral microbiota in *Rag1* ^*−/−*^ mice. Next, we asked if the age‐related decline in IgA production and associated changes in the oral microbiota were due to cellular senescence of the B cells. Since activated B cells in cervical lymph nodes expressing *Cdkn2a* in aged mice also express *p21* ^*Waf1/Cip1*^ (Figure S1b), inactivation of both *p16* ^*INK4a*^ and *p21* ^*Waf1/Cip1*^ is required to efficiently prevent the onset of cellular senescence in cervical lymph nodes (Takeuchi et al., 2010). However, since mice lacking both the *p16* ^*INK4a*^ and *p21* ^*Waf1/Cip1*^ genes (p16/p21‐double knockout (DKO) mice) begin to die of cancer at around 12 months of age and all die by 17 months (Kawamoto et al., 2023), it is impossible to fully age p16/p21‐DKO mice. We therefore used 15‐month‐old (middle‐aged) wild‐type (WT) and p16/p21‐DKO mice that had not yet developed cancer. B cells isolated from middle‐aged WT or p16/p21‐DKO mice were transplanted into *Rag1* ^*−/−*^ mice, along with T cells isolated from young WT mice (Figure 5a). In *Rag1* ^*−/−*^ mice transplanted with T cells from young mice and B cells from middle‐aged WT mice, IgA secretion was slightly restored, and changes in the oral microbiota after transplantation were small (Figure 5b,c). In contrast, in the *Rag1* ^*−/−*^ mice transferred with T cells from young WT mice and B cells from middle‐aged p16/p21‐DKO mice, IgA secretion was markedly restored in saliva (Figure 5b), and the composition of the oral microbiota was significantly altered after transplantation (Figure 5c). There were no significant differences in alpha diversity between the two groups after transplantation (Figure 5d). We then attempted to identify the oral bacteria that significantly changed after the transplantation with young T and middle‐aged p16/p21‐DKO B cells. We found that *S. nepalensis*, which was decreased with aging, dramatically increased in the mice transferred with young T and middle‐aged p16/p21‐DKO B cells (Figure 5e), as well as the mice transferred with young T and B cells (Figure 4e). *S. nepalensis* was found to be significantly increased in correlation with the reconstitution of functional IgA by transplantation, indicating that a decrease in salivary IgA secretion induced by B cell senescence causes a reduction of colonization of *S. nepalensis* in the oral cavity. Together, these results strongly suggest that cellular senescence of B cells cause a decrease in salivary IgA secretion and contribute to age‐related dysbiosis of the oral microbiota.

## 3 DISCUSSION

In recent years, it has become increasingly clear that the commensal microbiota plays an important role in maintaining host health, with particular attention being paid to the relationship between changes in the microbiota and aging. However, it is still unclear why the commensal microbiota changes with aging, especially in the oral microbiota. In this study, we focused on the role of cellular senescence in GC B cells in aging cervical lymph nodes. Unlike the gut, aging causes cellular senescence in oral GC B cells, regardless of the presence of commensal bacteria (Figures 1a–d and 2a–c). Note that follow‐up studies of age‐related changes in oral microbiota using the same mouse individuals (Figure 3) and transplantation experiments of B cells and T cells into *Rag1* ^*−/−*^ mice revealed a strong correlation between the age‐related decrease in salivary IgA secretion and the changes in oral microbiota (Figure 4a–e). Moreover, there is strong indication that one of the primary factors contributing to these alterations is the age‐related changes in B cells, which are dependent on senescence‐inducing genes *p16* ^*INK4a*^ and *p21* ^*Waf1/Cip1*^ (Figure 5a–e). Taken together, these results strongly suggest that age‐related induction of B cell senescence in cervical lymph nodes leads to alteration of the oral microbiota via a decrease in IgA secretion, resulting in age‐related alteration of the oral microbiota in mice.

What factors induce cellular senescence in GC B cells in cervical lymph nodes with aging? The oral cavity plays a crucial role as the frontline defense against viral infections (Sheikh‐Mohamed et al., 2022). It has been reported that upon infection with SARS‐CoV‐2 or influenza viruses, IgA antibodies are secreted in saliva to neutralize the viruses (Sterlin et al., 2021; Tsunetsugu‐Yokota et al., 2022), suggesting that viruses may also serve as inducers of IgA antibodies in saliva. Indeed, there is a report showing an association between the induction of IgA in saliva due to SARS‐CoV‐2 infection and changes in the oral microbiota (Soffritti et al., 2021). Moreover, it has been reported that SARS‐CoV‐2 infection can directly or indirectly induce cellular senescence (Camell et al., 2021; Delval et al., 2023; Gioia et al., 2023; Lee et al., 2021; Tsuji et al., 2022). Notably, the germ‐free mice utilized in our experiments are not devoid of viruses, thus precluding the complete exclusion of viral influenced on the host. In addition to exogenous viruses, age‐related expression of endogenous retroviruses is known to be one of the causes that induce cellular senescence (Liu et al., 2023). Mouse mammary tumor virus (MMTV) is known to exist as an active endogenous retrovirus in the mouse genome, and its expression has been reported to increase with age, triggering an inflammatory response (Liu et al., 2023). Indeed, the expression of MMTV‐encoded genes and proteins was found to increase with age in the cervical lymph nodes of both SPF and GF mice (Figure S4a,b). Thus, it is tempting to speculate that the accumulation of senescent cells observed in cervical lymph nodes may be due, at least in part, to chronic inflammation induced by age‐associated expression of endogenous retroviruses. On the other hand, epigenetic changes associated with aging in B cells of the oral immune system may contribute to the induction of cellular senescence. For example, downregulation of Bmi‐1, an epigenetic repressor of *p16* ^*INK4a*^ expression, reportedly increases the expression of *p16* ^*INK4a*^ in the submandibular gland (Yamakoshi et al., 2015) and B cells isolated from the bone marrow (Signer et al., 2008). In the present study, it was shown that the expression level of *Bmi‐1* in B cells in cervical lymph nodes decreases with age (Figure 1j). Hence, it is plausible that GC B cells in cervical lymph nodes undergo cellular senescence due to the upregulation of *p16* ^*INK4a*^ expression induced by the age‐related decline in *Bmi‐1* expression. Thus, it is possible that the cellular senescence of GC B cells in cervical lymph nodes with aging is at least partially due to the induction of *p16* ^*INK4a*^ expression by the age‐related decrease in *Bmi‐1* expression.

It is known that, as in the gut microbiota, the oral microbiota in humans also become imbalanced with aging (Willis et al., 2022). In addition, it has recently been reported that the ectopic colonization of oral bacteria, especially periodontopathic bacteria, can influence gut inflammation and age‐related diseases such as CRC (Atarashi et al., 2017; de Vos et al., 2022; Konishi et al., 2022; Konkel et al., 2019; Liao et al., 2024; Okumura et al., 2021), indicating that maintaining the proper balance of the oral microbiota is beneficial for the host. *S. nepalensis*, identified in this study as a bacterium that decreases with age (Figure 3h and Figure S3), is known to have antimicrobial activity against some pathogens (S. S. Singh et al., 2018). Therefore, *S. nepalensis*, one of the major constituent bacteria in young mice, may inhibit the colonization of other pathogenic bacteria in the oral cavity. Future studies need to clarify the impact of the colonization of *S. nepalensis* in the oral cavity on the host. On the other hand, given that the major genus constituting the oral microbiota differs between humans and mice (Segata et al., 2012), we need to identify oral bacteria regulated by IgA in humans, similar to those in mice.

In this study, we found that the frequency of IgA‐binding bacteria in saliva significantly increased with age (Figure 3g). Since we have shown that the specific binding of IgA to bacteria contributes to the maintenance of the gut microbiota (Kawamoto et al., 2014), age‐related changes in IgA binding to oral bacteria may also contribute to changes in the oral microbiota. IgA not only binds to pathogenic gut bacteria to eliminate them from the intestinal tract (Palm et al., 2014), but it is also known to promote the colonization of certain bacteria in the intestinal tract (Donaldson et al., 2018; Nakajima et al., 2018). Indeed, we have found that the abundance of *S. nepalensis* is positively correlated with IgA secretion (Figures 3h, 4e, and 5e, and Table S5), suggesting that binding of IgA to bacteria may contribute to the colonization of bacteria in the oral cavity. Future studies should examine in detail the change in specificity of IgA for oral bacteria with aging and the effect of IgA on the composition of the oral microbiota.

In addition to IgA, various factors in saliva have been implicated in the regulation of oral microbiota (Davenport, 2017). Age‐related changes in the salivary glands, which include infiltration of lymphocytes, fibrosis, and a decrease in the number of adenocytes, induce a reduction in saliva production (Li et al., 2023; Nelson et al., 2024; Yamakoshi et al., 2015). We found that tertiary lymphoid structures formed by infiltrated B cells in salivary glands with aging and that B cells located in these structures also expressed p16^INK4a^, suggesting that B cells in salivary glands also induce cellular senescence with aging (Figure S5a–d). Interestingly, B and T cells infiltrated in the salivary gland were identified as age‐associated B cells (ABC) and senescence‐associated T cells (SAT), which contribute to the formation of tertiary lymphoid structures (Bagavant et al., 2024; Kurosawa et al., 2021). In addition to these histological changes, our analysis of the aging process in the same individual mice also showed a significant decrease in saliva volume with aging (Figure S5e). Furthermore, it is known that not only the quantity but also the quality of saliva, such as the concentration of mucin and lactoferrin and peroxidase activity, declines with age (Xu et al., 2019). Therefore, as a limitation of this study, we cannot rule out the possibility that age‐related changes in salivary glands influence changes in the oral microbiota. However, B cell transplantation experiments using *Rag1* ^*−/−*^ mice have revealed that age‐related B cell senescence decreases IgA secretion and contributes, at least to some extent, to changes in the oral microbiota. Therefore, identifying stress sources that induce cellular senescence in GC B cells within cervical lymph nodes will aid in preventing age‐related changes in the oral microbiota. This, in turn, may help suppress various diseases caused by the ectopic growth of oral bacteria.

## 4 CONCLUSIONS

In the present study, we found that the induction of cellular senescence of GC B cells in cervical lymph nodes with aging causes a decrease in IgA secretion and induces age‐related changes in the oral microbiota. These findings expand our understanding of how changes in the oral microbiota are related to aging and open new possibilities for their control.

## 5 MATERIALS AND METHODS

### 5.1 Mice

Albino *p16‐luc* mice (Yamakoshi et al., 2009) were generated by crossing with C57BL/6 albino mice (The Jackson Laboratory Japan) for six generations. Then, the frozen embryos were created using seventh generation of *p16‐luc* male mice and C57BL/6 albino female mice. All germ‐free *p16‐luc* mice used for this experiment were generated from these embryos. Around the same time, *p16‐luc* male mice from the seventh, eighth, and ninth generations were crossed with C57BL/6 albino female mice to produce SPF *p16‐luc* mice. These mice were housed in SPF facilities at the Research Institute for Microbial Diseases (RIMD) at Osaka University or germ‐free facilities at the Central Institute for Experimental Animals (CIEA) after sterilization.

Wild‐type (WT) C57BL/6 mice were purchased from CLEA Japan and The Jackson Laboratory Japan. C57BL/6 Albino *p16‐Luc* mice, p16/p21‐DKO mice (Takeuchi et al., 2010), *Rag1* ^*−/−*^ mice, and WT mice were bred and reared in the SPF facility at RIMD at Osaka University. The number and age of mice used for the experiments were represented in the figure captions. Mice were bred and reared at 23 ± 2°C with a humidity of 55 ± 15% and on a 12 h light/12 h dark cycle and fed a normal diet (CE‐2 from CLEA Japan; sterilized 20 kGy radiation exposure). Detailed breeding conditions and mouse information used in Figure 3 are summarized in Tables S1 and S2, respectively.

### 5.2 Ethics approval

All mouse experiments were approved by the Animal Research Committee of the Research Institute for Microbial Diseases (RIMD) at Osaka University and the Central Institute for Experimental Animals (CIEA).

### 5.3 Bioluminescence imaging

For noninvasive BLI, the mice were anesthetized with isoflurane and injected intravenously with 75 mg/kg D‐luciferin substrate (126–05116; Fujifilm Wako Pure Chemicals) (30 mg/mL in saline), and imaging was performed 5 min later. For ex vivo imaging, mice were anesthetized by intraperitoneal injection of three kinds of anesthetics (medetomidine (75 μg/mL; Zenoac), midazolam (400 μg/mL; Sandoz), and butorphanol (500 μg/mL; Meiji Seika Pharma)) and injected intravenously with 75 mg/kg D‐luciferin substrate (30 mg/mL in saline). Then, the mice were euthanized 5 min later, and the collected organs were used for imaging. Imaging was performed using an IVIS Lumina XRMS Series III (Perkin Elmer) with “medium” as binning and 5 min for exposure time. Bioluminescence imaging data were analyzed with Living Image Software (ver. 3.0; Perkin Elmer).

### 5.4 Quantitative real‐time PCR analysis

Total RNA extraction from cells and tissues was performed with the RNeasy Mini kit (74106; Qiagen) and TRIzol (15596018; Thermo Fisher Scientific) according to the manufacturer's protocol. Genomic DNA removal and complementary DNA (cDNA) synthesis were performed with the PrimeScript RT reagent Kit with a gDNA Eraser (RR047A; Takara Bio). RT‐qPCR was performed with TB Green Premix Ex Taq II (RR820A; Takara Bio) on Thermal Cycler Dice Real Time System III (Takara Bio). The messenger RNA expression level in each gene was calculated relative to the β‐actin expression level. The sequences of PCR primer used for the experiment are shown in Table S3.

### 5.5 Cell isolation and sorting

Cells from the spleen and mesenteric lymph nodes (MLNs) were isolated as described previously (Kawamoto et al., 2014). Cells from the cervical lymph nodes (CLNs) were prepared as described below. The CLNs collected from mice were smashed using slide glasses and filtrated with the mesh to remove debris. For RT‐qPCR analysis, B220^+^PNA^+^ and B220^+^PNA^−^ in CLNs were collected as GC B cell and non‐GC B cell, respectively, using a cell sorter (SH800S; Sony). Collected cells were suspended in TRIzol (15,596,018; Thermo Fisher Scientific) and kept at −80°C until use. For scRNAseq analysis, CD45^+^ cells were sorted with a SH800S (Sony). For transfer experiments, cells isolated from spleen, MLNs, and CLNs from WT and p16/p21‐DKO mice were depleted of B220^+^ or CD4^+^ cells by IMag cell separation system (Becton Dickinson) for T or B cell sorting, respectively. The recovered cells were further stained with antibodies against CD4 or B220 for T or B cells, respectively, and sorted with a SH800S (Sony). The purity of sorted cell populations was constantly >99.5%. The following antibodies were used in the surface staining for sorting: PE‐conjugated‐anti‐mouse/human B220 (1:100; 103207; BioLegend), Biotinylated PNA (1:800; B‐1075‐5; Vector Laboratories), APC‐conjugated‐streptavidin (1:100; 405207; BioLegend), APC‐conjugated‐anti‐mouse CD45 (1:100; 103111; BioLegend), and PE‐conjugated anti‐mouse CD4 (1:100; 100512; BioLegend).

### 5.6 Single‐cell RNA‐seq analysis

The single‐cell suspension was processed on a Chromium Controller (10× Genomics) according to the Chromium Single Cell 3′ Reagent Kits protocol. For this process, the following reagents were used: Chromium Next GEM Single Cell 3′ GEM, Library & Gel Bead Kit v3.1 (PN‐1000128; 10× Genomics), Chromium Next GEM Chip G Single Cell Kit (PN‐1000127; 10× Genomics), and Single Index Kit T Set A (PN‐1000213; 10× Genomics). For library preparation and sequencing, about 16,500 live cells per sample were loaded onto the Chromium Controller to produce 10,000 single‐cell gel‐bead emulsions. To create cDNA tagged with a cell barcode and unique molecular identifier (UMI), Oil droplets containing barcode beads (GEM) and single‐cell were reverse transcribed in a Veriti Thermal Cycler (Thermo Fisher Scientific). Single‐cell libraries were generated by amplifying cDNA according to the manufacturer's protocol and quantified by an Agilent Bioanalyzer High Sensitivity DNA assay using a High Sensitivity DNA Kit (5067–4626; Agilent). Then, they were fragmented enzymatically, end repaired, and tagged by polyA. Clean‐up and size selection of amplified cDNA were processed using SPRIselect magnetic beads (B23317; Beckman Coulter). The size‐selected fragments were ligated by Illumina sequencing adaptors. After that, the ligated fragments were cleaned up using SPRIselect magnetic beads. Finally, sample indices were selected and amplified by double‐sided size selection using SPRIselect magnetic beads. The Agilent Bioanalyzer High Sensitivity DNA assay was used to check the quality of the final library. Sequencing of the samples was performed on an Illumina NovaSeq 6000 in paired‐end mode (read 1: 28 base pairs (bp); read2: 91 bp). Aligning of the raw sequencing reads to the mouse reference genome mm10 was performed using the Cell Ranger (version 7.1.0) analysis pipeline (10x Genomics) and then UMI count matrices were created. Seurat (version 4.3.0.1) was used for the generated matrices and downstream analysis. Cells and genes listed below were excluded from the analysis: cells including <200 expressed genes; and genes found in fewer than three cells. the doublets were excluded from the analysis by DoubletFinder (version 2.0.3). Then, sctransform (Choudhary & Satija, 2022) was performed for the normalization and variance stabilization. Some genes with low expression levels (*Cdkn2a, Cdkn1a*) were analyzed using count data. Principal component analysis was performed using the dataset's top 3000 highly variable genes for dimensionality reduction. Then, Clusters were decided using the 1st to 30th principal components and shared nearest neighbor graphs. Visualization of clusters was performed using uniform manifold approximation and projection (UMAP).

### 5.7 Immunohistochemistry

The tissues were fixed using Bouin's fixative (33142; Muto Pure Chemicals) for 2 h at room temperature and embedded in paraffin. Paraffin blocks were sectioned at 5 μm. The sections were deparaffinized using PathoClean (161–28321; FUJIFILM Wako Pure Chemical Corporation) and blocked with a BIOXALL Endogenous Blocking Solution (SP‐6000; Vector Laboratories) and further blocked with 5% horse serum for 30 min at room temperature. The primary antibodies used were as follows: anti‐mouse p16^INK4a^ (1:1000; ab211542; Abcam), biotin‐labeled anti‐mouse/human B220 (1:100; 103203; Biolegend), and anti‐γH2A.X (phospho S139) (1:1000, Abcam, ab11174). The secondary antibody and substrate were used as follows: ImmPRESS HRP Horse Anti‐Rabbit IgG Polymer Detection Kit (MP‐7401; Vector Laboratories) and TSA Plus TMR kit (NEL742001KT; Akoya Bioscience) or Alexa Fluor Plus 555 donkey anti‐Rabbit IgG (1:1000; A32794; Thermo Fisher Scientific) for p16^INK4a^ staining; Alexa Fluor 488 donkey anti‐rat IgG (1:1000; A21208; Thermo Fisher Scientific) for B220 staining; ImmPRESS HRP Horse Anti‐Rabbit IgG Polymer Detection Kit and TSA plus Cyanine 5 kit (NEL745001KT; Akoya Biosciene) for γH2A.X staining. For costaining of γH2A.X and p16^INK4a^, γH2A.X was stained first, then treated with microwave oven heating in 10 mM citrate buffer (pH 6.0) for 20 min for antibody stripping, and p16^INK4a^ staining was followed. Nuclear staining was performed using DAPI (D523; DOJINDO) and reacted with TrueBlack Lipofuscin Autofluorescence Quencher (23007; Biotium). The sections were mounted using Fluoromount‐G (0100–01; Southern Biotech). Fluorescence observation was performed using a BZ‐X710 fluorescence microscope (Keyence). The detection of mouse mammary tumor virus (MMTV) was followed by the literature (Liu et al., 2023). The tissues were fixed using paraformaldehyde (162–16065; FUJIFILM Wako Pure Chemical Corporation) for 24 h at 4°C and embedded in paraffin. The sections were deparaffined and blocked as described above. Rabbit polyclonal anti‐MMTV‐Env (1:1000; NBP2‐44179; Novus Biologicals) and biotin‐conjugated anti‐Rabbit IgG (BA‐1000; Vector Laboratories, Inc.) were used as a primary and secondary antibodies, respectively. After incubation with VECTASTAIN® Elite® ABC Kit Peroxidase (PK‐6100; Vector Laboratories, Inc.), the signal was detected with DAB Substrate Kit (34002, ThermoFisher). Nuclear staining was performed using hematoxylin (30002, Muto Pure Chemicals). The sections were mounted using PARAmount‐N (308–400‐1, FALMA). Images were captured using a BZ53 microscope (Olympus).

### 5.8 Proliferation assay in vivo

Young (3‐month‐old) or aged (20‐month‐old) WT mice were injected intraperitoneally with EdU (4 mg/mL in saline; 050–08844; FUJIFILM Wako Chemicals) every 2 days. Mice were sacrificed to collect of tissues 14 days after the first injection. Click‐iT Plus EdU Alexa Fluor 647 imaging kit (C10640; Thermo Fisher Scientific) was used for the detection of EdU incorporation. The images, including B cell populations, were taken randomly and used for the count of EdU^+^ B cells. The number of EdU^+^ cells was counted in p16^INK4a^‐positive or ‐negative B cells, and the frequency of EdU^+^ cells in each B cell population was calculated.

### 5.9 Collection of saliva

Saliva was collected as described below. Mice were anesthetized by intraperitoneal injection of three kinds of anesthetics (medetomidine (75 μg/mL), midazolam (400 μg/mL), and butorphanol (500 μg/mL)), and injected intraperitoneally with 1 mg/kg pilocarpine (28008–31; Nacalai Tesque). One minute later, saliva was collected for 10 min and kept at −80°C. The total volume of saliva was measured. Saliva was collected from mice in the morning without fasting.

### 5.10 IgA ELISA

The supernatant of saliva was used for IgA ELISA. IgA ELISA was performed as follows. Clear Flat‐Bottom Immuno Nonsterile 96‐Well Plates (442404; Thermo Fisher Scientific) were coated with anti‐mouse IgA antibody (1:1000; A90‐103A; Bethyl Laboratories) for 1 h at room temperature and then blocked with 5% BSA for 30 min at room temperature. The coated plate was reacted with samples for 1 h at room temperature followed with the incubation with HRP‐conjugated anti‐mouse IgA (1:1000; A90‐103P; Bethyl Laboratories) for 1 h at room temperature. The plate was incubated for 30 min at room temperature after adding 3,3′,5,5′‐Tetramethylbenzidine liquid substrate (152346; MP Biomedicals), and finally, the reaction was stopped using 2 M H_2_SO4. The absorbance OD450 was evaluated using BioTek Synergy HTX (Agilent). The IgA concentration of the samples was calculated from the standard value measured by mouse IgA isotype control antibody (0106–01; SouthernBiotech) and the dilution of the sample. The IgA secretion was calculated using the saliva volume and the IgA concentration.

### 5.11 Analysis of IgA bound bacteria

Saliva was centrifuged (15,000 g, 10 min, 4°C) and the pellet was suspended with 1% BSA/5% goat serum/ PBS (on ice, 10 min) for the blocking. Then, pellets were stained with PE‐conjugated‐anti‐mouse IgA (1:100; 1040–09; SouthrnBiotech) (on ice, 15 min) and DAPI (on ice, 30 min). IgA‐positive or ‐negative bacteria were analyzed by a cell sorter. Analysis of flow cytometry data was performed on FlowJo version 10.8.1 software (Beckton Dickinson).

### 5.12 DNA extraction from bacteria

DNA extraction from saliva samples was performed using PureLink™ Genomic DNA Mini Kit (K182002; Thermo Fisher SCIENTIFIC) according to the manufacture's protocol.

### 5.13 16S rRNA gene sequencing

16S rRNA gene sequencing was performed as previously described (Kawamoto et al., 2023). Amplifying the V1‐V2 region of the bacterial 16S rRNA gene was performed using KAPA HiFi HotStart ReadyMix (Roche) with universal 16S rRNA primers. Then, PCR fragments were added with the Illumina flow cell adaptors and indices by secondary PCR amplification. The sequences of PCR primer used for the experiment are shown in Table S3. Illumina MiSeq platform (Illumina) was used for 16S rRNA gene sequencing with a MiSeq Reagent Kit V2 (Illumina) (paired end; 250 cycles × 2). QIIME 2 (version 2020.8) (Bolyen et al., 2019) pipeline was used for analysis of sequencing reads. Denoise was performed with the DADA2 plugin (Callahan et al., 2016) and fastq files were counted as amplicon sequence variants (ASVs). Naïve Bayes classifier trained on the SILVA 16S rRNA sequence database (version 138) in the QIIME 2 pipeline was used for phylogenetically classifying ASVs. The alpha diversity (Faith's phylogenetic diversity) was calculated using a phylogenetic tree. Beta diversity (principal coordinate analyses of the Bray‐Curtis distance) was calculated with a sampling depth of 10,000 reads. The cross‐nested differential abundance analysis was performed using R software version 4.1.0 with the MaAsLin2 package, which could evaluate multivariable association between the age and the microbial features adjusted by covariates such as mouse identity, sex, cage and parents (Mallick et al., 2021). We set the minimum value of the genus prevalence (min_prevalence) to 0.1 and the maximum q value (max_significance) to 0.25 for parameter setting. The genus with significant age‐related change detected by MaAsLin2 were shown in Figure 3e,f. The ASVs with significant age‐related change detected by MaAsLin2 were shown in Figure 3h. The bacteria that significantly changed in the transferred mice depending on functional IgA in Figures 4e, and 5e were identified as follows. First, we compared the relative frequency of ASVs before and after the transplantation in the mice transferred with young T and B cells or the mice transferred with young T cells and mid‐aged p16/p21‐DKO B cells using Wilcoxon matched‐paired signed rank test and identified ASVs, which are significantly changed in each mouse after transplantation. Furthermore, we compared the relative frequency of each ASV after transplantation between the mice transferred with young T and B cells and aged T and B cells, or the mice mid‐aged WT B cells and p16/p21‐DKO B cells using the Wilcoxon rank‐sum test, and identified ASVs which are significantly changed in transplanted mice depending on functional IgA. The heatmaps of the ASVs (Figures 3f, h, 4e, and 5e) were created using Heatmapper (http://www.heatmapper.ca/). We calculated the standardization value of ASV by subtracting the relative abundance of ASV before and after transplantation (Figures 4e, and 5e).

### 5.14 Adoptive cell transfer experiments

The procedure to isolate T cells and B cells were described in “Cell isolation and sorting.” For the transfer experiment using T cells and B cells, 2 × 10^7^ cells isolated from spleen, MLN, and CLNs of young (3‐month‐old) or aged (20‐month‐old) WT mice were injected intravenously into *Rag1* ^*−/−*^ mice. For the cotransfer experiment, B cells were isolated from the spleen, MLNs, and CLNs of aged WT or p16/p21‐DKO mice (15 months old). T cells were isolated from the spleen, MLNs, and CLNs of young WT mice (3 months old). The mixture of 1 × 10^6^ CD4^+^ T cells and 3 × 10^6^ B220^+^ B cells was injected intravenously into *Rag1* ^*−/−*^ mice. The recipient mice were analyzed 3–5 months after the transplantation.

### 5.15 Flow cytometry

The salivary gland tissue was minced and treated with 2 mg/mL Collagenase type II (LS004176; Worthington Biochemical) and 1 mg/mL DNase I (D5025; Sigma) for 25 min at 37°C. Then, the digested sample was incubated for 10 min at 37°C after the supplementation of 0.5 M EDTA at 10 μL per 1 mL volume. The cell debris were removed by Percoll (17089101; Cytiva) and isolated cells were stained with reagents and antibodies as follows. Approximately 2 × 10^6^ cells were first stained with Zombie Yellow Fixable Viability Kit (1:1000 in PBS, L34967; BioLegend) following manufacture's protocol. Then, these cells were blocked with 5% normal rat serum. The following antibodies were used in the surface staining for B cell analysis: APC‐conjugated‐anti‐mouse/human B220 (1:100; 103211; Biolegened). The stained cells were filtrated with a cell strainer (352235; Beckton Dickinson) and analyzed using SH800S (Sony). Analysis of flow cytometry data was performed on FlowJo version 10.8.1 software (Beckton Dickinson).

### 5.16 Statistics

Almost all data were analyzed and visualized using Prism (version 10.0.3). Figures 3b, h, 4e, 5e and Figure S5e were analyzed with R (version 4.1.0). Figures 3c,d, 4c,d, 5c,d were analyzed with the QIIME 2 (version 2020.8) pipeline. Statistical significance was determined by two‐tailed unpaired *t* test (Figures 1i, j, 4b, and 5b), two‐tailed paired *t* test (Figure 3g), two‐way analysis of variance (ANOVA) followed by Šídák's multiple comparisons test (Figure 1c,d, Figure S4a), Mann–Whitney *U* test (Figure 2b, Figure S5c,d), Friedman rank‐sum test followed by pairwise Wilcoxon signed rank test (Figure 3b), Wilcoxon matched‐paired signed‐rank test (Figures 3h, 4e, and 5e), Wilcoxon rank‐sum test (Figures 4e and 5e), one‐way analysis of variance (ANOVA) followed by pairwise two‐tailed paired *t* test (Figure S5e), Spearman's rank correlation coefficient (Tables S4 and S5). Aged mice that developed cancer were excluded from the analysis, and data obtained from the mice that developed cancer were excluded from the analysis.

## AUTHOR CONTRIBUTIONS

S.K. and E.H. designed the experiments, and wrote the manuscript. H.M., S.K., K.U, J. H. P., N.H., and Y.O. performed the experiments and meta 16S rRNA gene‐sequencing analysis. H.M. and Y.K. performed scRNAseq analysis. All the authors discussed the results and commented on the manuscript.

## FUNDING INFORMATION

This work was supported in part by grants from the Japan Agency for Medical Research and Development under grant numbers JP21gm5010001h0005, JP22gm1710004h0001, JP22zf0127008h0001 and JP22ama221114h0001 (to E.H.), the Japan Science and Technology Agency under grant numbers JPMJMS2022 (to E.H.), the Japan Society for the Promotion of Science under grant numbers JP20K07446, JP23K06481 (to S.K.), and JP22H00457 (to E.H.), the Princess Takamatsu Cancer Research Fund, Kato Memorial Bioscience Foundation, Naito Foundation, UBE Foundation, and Takeda Science Foundation (to S.K.), and the Mitsubishi Foundation (to E.H.). Some of the aged mice were provided by the Foundation for Biomedical Research and Innovation at Kobe through the National BioResource Project of the Ministry of Education, Culture, Sports, Science and Technology in Japan (to S.K.).

## CONFLICT OF INTEREST STATEMENT

None declared.

## Supporting information


Table S1.

Appendix S1.

## Data Availability

16S rRNA gene sequence analysis was performed using the SILVA 16S rRNA sequence database (version 138) (https://www.arb‐silva.de/). The datasets (the bacterial 16S rRNA gene sequence and scRNAseq data) supporting the conclusions of this article are available in the DNA Data Bank of Japan (https://www.ddbj.nig.ac.jp) with the accession codes PRJDB17492 and PRJDB17493, PRJDB17494, respectively. In addition, processed data for scRNAseq have been deposited in the DNA Data Bank of Japan with the accession code E‐GEAD‐676.
