# Supplementary material for: B cell senescence promotes age‐related changes in oral microbiota
Source: Aging Cell. 2024 Aug 9;23(12):e14304. doi: 10.1111/acel.14304 (PMC11634744; doi:10.1111/acel.14304)
Supplement: Supplementary file 1 — Table S1. [file ACEL-23-e14304-s002.pdf]

Table S1. Mouse breeding condition

GF	Manufacture	Product name	Other information
Feed	CLEA Japan	CLEA Rodent Diet CL-2	Radiation-sterilized diets (20 kGy)<br>Before transfering into a vinyl isolater, diet is autoclaved.
Water			Sterilized tap water by autoclave
Bedding	CLEA Japan	CL-4161 Clean Chips	
Cage	CLEA Japan	CM-3 Mouse Cage	Dimension (WxHxD (mm)): 125 x110 x195<br>Housing density is up to 5 mouse per cage

SPF	Manufacture	Product name	Other information
Feed	CLEA Japan	CLEA Rodent Diet CE-2	Radiation-sterilized diets (20 kGy)
Water	Avidity Science	Automatic water supply system Edstrom	Tap water is filtered using PENTEK (1um, Avidity Science) and irradiated with UV, then chlorine is added at a concentration of about three ppm.
Bedding	Japan SLC	Paper Clean	
Cage	TECHNIPLAST	GM500SealSafePlus cage	Indivisually ventilated cage sysem<br>Dimension (WxHxD (mm)): 199 x131 x379<br>Housing density is up to 7 mouse per cage

Table S2. Mouse information used for Fig. 3.

Mouse ID	Sex	Cage	Parents
1	Male	Cage1	A
2	Male	Cage1	A
3	Male	Cage1	A
4	Male	Cage2	C
5	Male	Cage2	D
6	Male	Cage2	D
7	Male	Cage2	E
8	Male	Cage2	E
9	Male	Cage2	E
10	Male	Cage3	E
11	Male	Cage3	C
12	Male	Cage3	D
13	Female	Cage4	F
14	Female	Cage4	A
15	Female	Cage4	A
16	Female	Cage4	F
17	Female	Cage5	D
18	Female	Cage6	D
19	Female	Cage6	C
20	Female	Cage6	C
21	Female	Cage6	E
22	Female	Cage7	G
23	Female	Cage7	G
24	Female	Cage7	G

Table S3. Primers used in this study

**Primers List for 16S rRNA gene-sequencing**		
1st PCR		
Primer name	Forward or Reverse	Sequence
27F	Forward	ACACTCTTTCCCTACACGACGCTCTTCCGATCTNN AGRGTTTGATYMTGGCTCAG
338R	Reverse	GTGACTGGAGTTCAGACGTGTGCTCTTCCGATCTN NTGCTGCCTCCCGTAGGAGT
2nd PCR		
Primer name	Forward or Reverse	Sequence
D501	Forward	AATGATACGGCGACCACCGAGATCTACACTATAGC CTACACTCTTTCCCTACACGACGCTCTTCCGATCT
D502	Forward	AATGATACGGCGACCACCGAGATCTACACATAGA GGCACACTCTTTCCCTACACGACGCTCTTCCGATCT
D503	Forward	AATGATACGGCGACCACCGAGATCTACACCCTATC CTACACTCTTTCCCTACACGACGCTCTTCCGATCT
D504	Forward	AATGATACGGCGACCACCGAGATCTACACGGCTC TGAACACTCTTTCCCTACACGACGCTCTTCCGATCT
D505	Forward	AATGATACGGCGACCACCGAGATCTACACAGGCG AAGACACTCTTTCCCTACACGACGCTCTTCCGATC T
D506	Forward	AATGATACGGCGACCACCGAGATCTACACTAATCT TAACACTCTTTCCCTACACGACGCTCTTCCGATCT
D507	Forward	AATGATACGGCGACCACCGAGATCTACACCAGGA CGTACACTCTTTCCCTACACGACGCTCTTCCGATCT
D508	Forward	AATGATACGGCGACCACCGAGATCTACACGTACTG ACACACTCTTTCCCTACACGACGCTCTTCCGATCT
D701	Reverse	CAAGCAGAAGACGGCATACGAGATCGAGTAATGT GACTGGAGTTCAGACGTGTGCTCTTCCGATCT
D702	Reverse	CAAGCAGAAGACGGCATACGAGATTCTCCGGAGT GACTGGAGTTCAGACGTGTGCTCTTCCGATCT
D703	Reverse	CAAGCAGAAGACGGCATACGAGATAATGAGCGGT GACTGGAGTTCAGACGTGTGCTCTTCCGATCT
D704	Reverse	CAAGCAGAAGACGGCATACGAGATGGAATCTCGT GACTGGAGTTCAGACGTGTGCTCTTCCGATCT
D705	Reverse	CAAGCAGAAGACGGCATACGAGATTTCTGAATGT GACTGGAGTTCAGACGTGTGCTCTTCCGATCT
D706	Reverse	CAAGCAGAAGACGGCATACGAGATACGAATTCGT GACTGGAGTTCAGACGTGTGCTCTTCCGATCT
D707	Reverse	CAAGCAGAAGACGGCATACGAGATAGCTTCAGGT GACTGGAGTTCAGACGTGTGCTCTTCCGATCT
D708	Reverse	CAAGCAGAAGACGGCATACGAGATGCGCATTAGT GACTGGAGTTCAGACGTGTGCTCTTCCGATCT
D709	Reverse	CAAGCAGAAGACGGCATACGAGATCATAGCCGGT GACTGGAGTTCAGACGTGTGCTCTTCCGATCT
D710	Reverse	CAAGCAGAAGACGGCATACGAGATTTCGCGGAGT GACTGGAGTTCAGACGTGTGCTCTTCCGATCT
D711	Reverse	CAAGCAGAAGACGGCATACGAGATGCGCGAGAG TGACTGGAGTTCAGACGTGTGCTCTTCCGATCT
D712	Reverse	CAAGCAGAAGACGGCATACGAGATCTATCGCTGT GACTGGAGTTCAGACGTGTGCTCTTCCGATCT
**Primers List for quantitative real-time PCR**		
Gene name	Forward or Reverse	Sequence
mouse *b-actin*	Forward	GATGACCCAGATCATGTTTGA
mouse *b-actin*	Reverse	GGAGAGCATAGCCCTCGTAG
mouse *$p16^{INK4a}$*	Forward	GAACTCTTTCGGTCGTACCC
mouse *$p16^{INK4a}$*	Reverse	CGAATCTGCACCGTAGTTGA
mouse *Bmi-1*	Forward	TTTTATGCAGCTCACCCGTC
mouse *Bmi-1*	Reverse	TCACCTCTTCCTTAGGCTTCTC
mouse *MMTV*	Forward	GATGGTATGAAGCAGGATGG
mouse *MMTV*	Reverse	AAGGGTAAGTAACACAGGCAGATGTA

Table S4. Correlation between IgA secretion and each genus

Genus	Spearman's rho	p value	Significant difference
Streptococcus	-0.072768942	0.42908246	
Staphylococcus	0.297319258	0.0010214	***
Jeotgalicoccus	0.346450076	0.0001061	****

* $p < 0.05$, ** $p < 0.01$, *** $p < 0.005$, **** $p < 0.001$.

Table S5. Correlation between IgA secretion and each species

Species	ASV	Spearman's rho	p value	Significant difference
Streptococcus	a73cc4d7d4161951b9077aae10e26224	0.403492966	4.89E-06	****
Streptococcus	7811c97cf7305669abb55a95a77b71fd	0.193395375	0.034457447	*
Streptococcus	4d63b9a67784f8a56c6c8aa1de2beaa6	-0.085054518	0.355167011	
Staphylococcus nepalensis	6c265c5d851ad46ace2eb74d2f6143f6	0.393298373	8.84E-06	****
Staphylococcus nepalensis	c4ce89441f3d5126e8f4e0ac0b2c966f	0.351037552	8.46E-05	****
Staphylococcus nepalensis	2a630be08802ee9ccf23c08ae9a68140	0.293499541	0.001140187	***
Staphylococcus	bd5206138a28a0d6b8cacfaaca2999a1	0.167158602	0.068027265	
Staphylococcus pasteuri	0075f3526d9a408eb64a43ad9e41d625	0.032792265	0.722172368	
Staphylococcus	2baaef35cddaa96b8280849cd6f92023	0.234553318	0.009920552	**
Staphylococcus epidermidis	4bbb968c681ad87b556889c92e01bc6f	-0.038614746	0.675411975	
Staphylococcus sciuri	59d389ffe3006af1b6807aa6b2ec9a26	0.371952162	2.88E-05	****
Staphylococcus sciuri	246bbfeea1a2f2998be0feaa0ffcf831	0.543596902	1.40E-10	****
Jeotgalicoccus	53c34e79c9b40e79812b558a1f2df1ca	0.464374522	9.17E-08	****
Jeotgalicoccus	eb5a8a0e015f6d59c739632c4b1c89ac	0.419946597	1.80E-06	****
Jeotgalicoccus	badc0e9a86683a0fc43a6c14eaa546a0	0.359831439	5.42E-05	****
Jeotgalicoccus	efa82071ffb88dd767949106d5c0b693	0.377482063	2.14E-05	****
Jeotgalicoccus	46a8c4ede409c3e9a9c0b247e28e08ae	0.38062182	1.80E-05	****
Jeotgalicoccus	6c0a8d6a980b68f94cd152f220aeff9d	0.095169728	0.30115063	
Jeotgalicoccus	97b34d42ee2a6e5af0e6f1a2f63eefec	-0.03401172	0.712286722	

* $p < 0.05$, ** $p < 0.01$, *** $p < 0.005$, **** $p < 0.001$.
