# Supplementary material for: B cell senescence promotes age‐related changes in oral microbiota
Source: Aging Cell. 2024 Aug 9;23(12):e14304. doi: 10.1111/acel.14304 (PMC11634744; doi:10.1111/acel.14304)
Supplement: Supplementary file 2 — Appendix S1. [file ACEL-23-e14304-s001.docx]

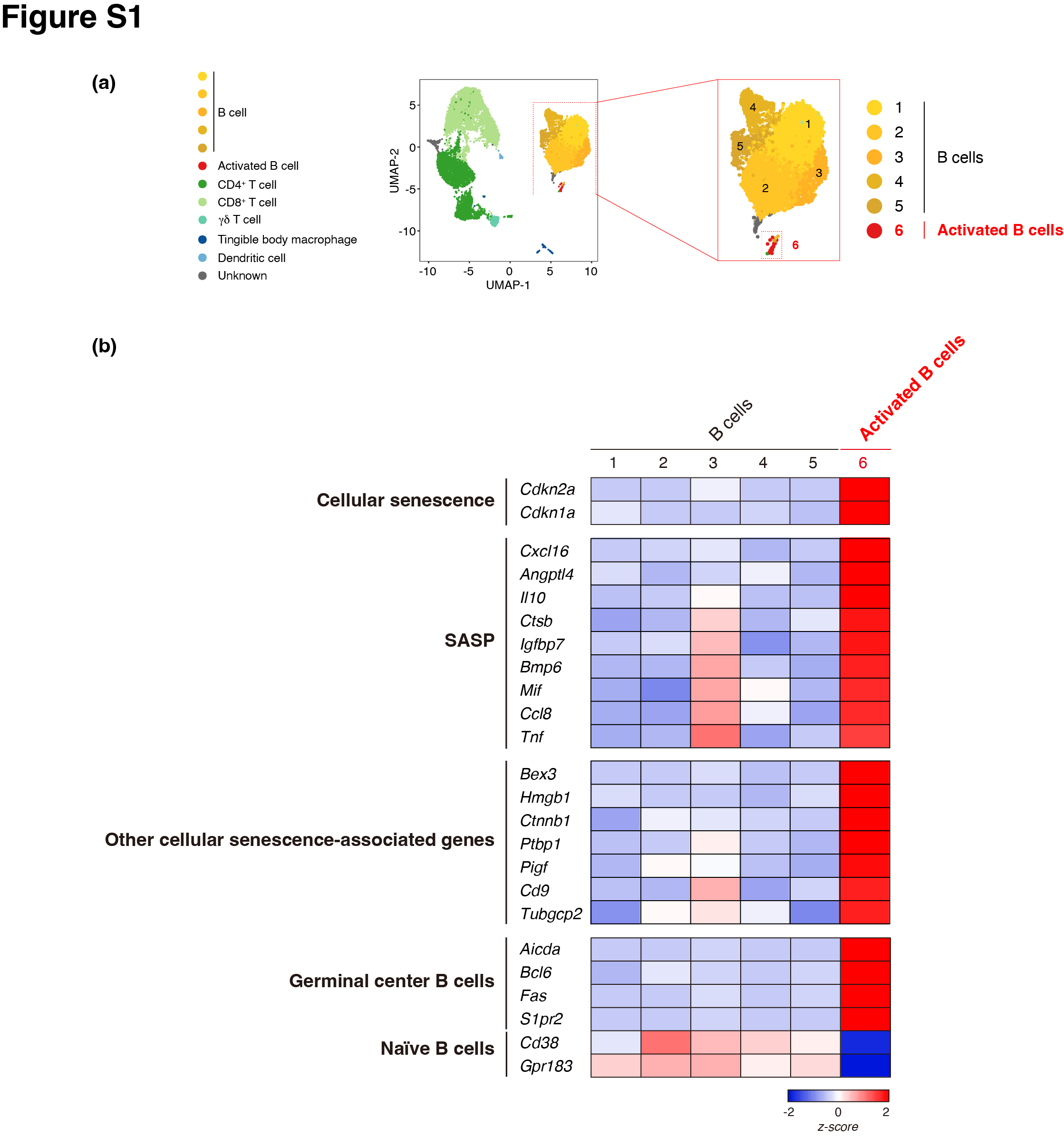


**Figure S1. Expression of senescence-associated genes in GC B cells in cervical lymph nodes of aged mice**

**(a)** UMAP plot gathering all CD45^+^ cells from cervical lymph nodes from 6 or 20M SPF WT mice, in which all cells are clustered and color-coded by cell types (**left panel**). B cells and activated B cells, which are enclosed by the red dotted line, were extracted and subdivided into six clusters (**right panel**). **(b)** Heat map showing differential expression levels of genes classified as markers of cellular senescence, SASP factors, and markers of germinal center or naïve B cells in these B-cell clusters. Negative or positive z-scores are indicated as blue or red intensity, respectively. Note that activated B cells (cluster #6), in which cells with high expression of *Cdkn1a* and *Cdkn2a* are present, show the phenotype of germinal center B cells and highly express a series of SASP factors.


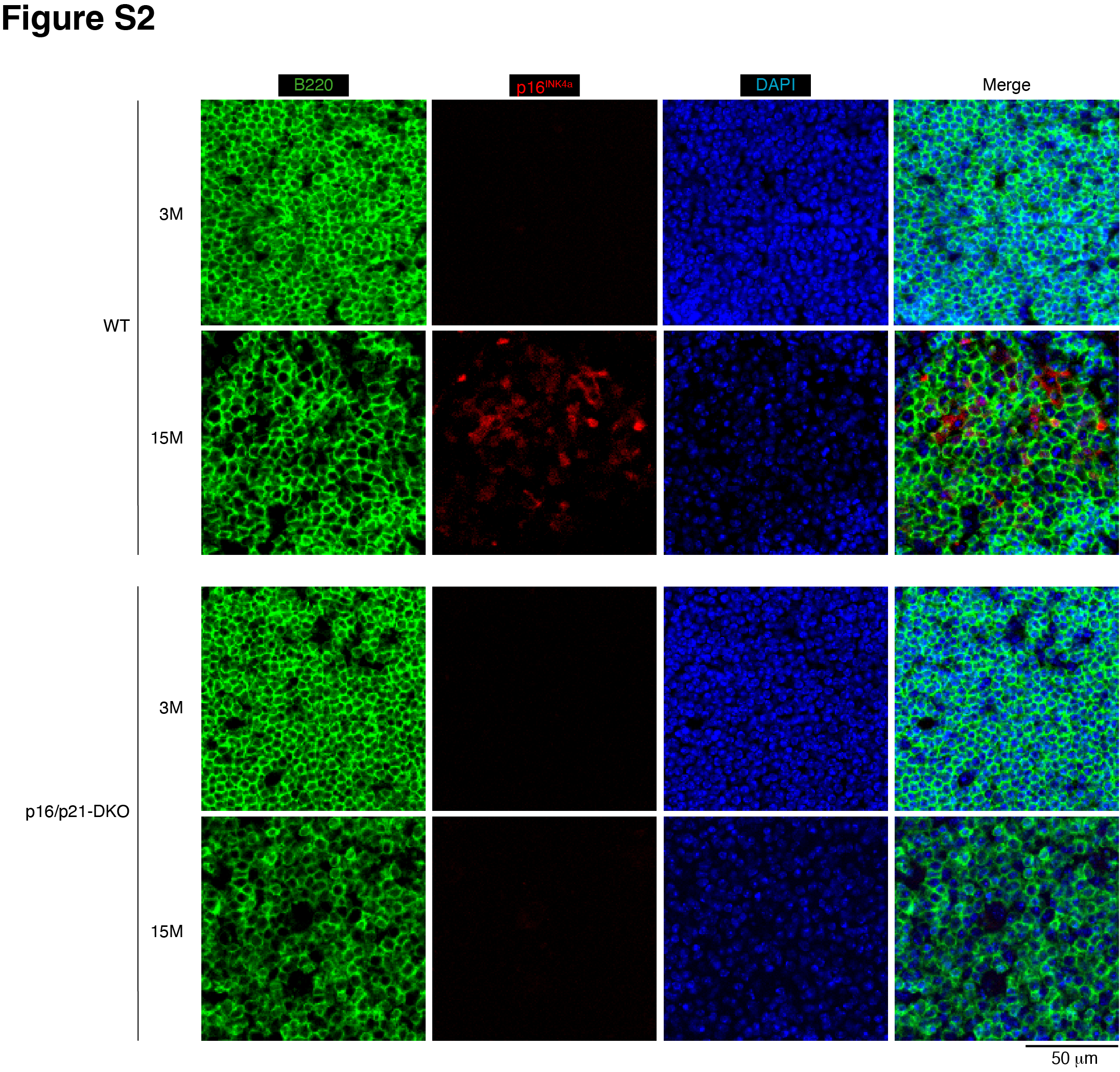


**Figure S2. Confirmation of an immunohistochemical p16^INK4a^ staining in aged mice**

Immunohistochemical images of cervical lymph nodes from 3- or 15-month-old WT and p16/p21-DKO mice (male) stained with antibodies against B220 and p16^INK4a^.

Scale bar, 50 μm.


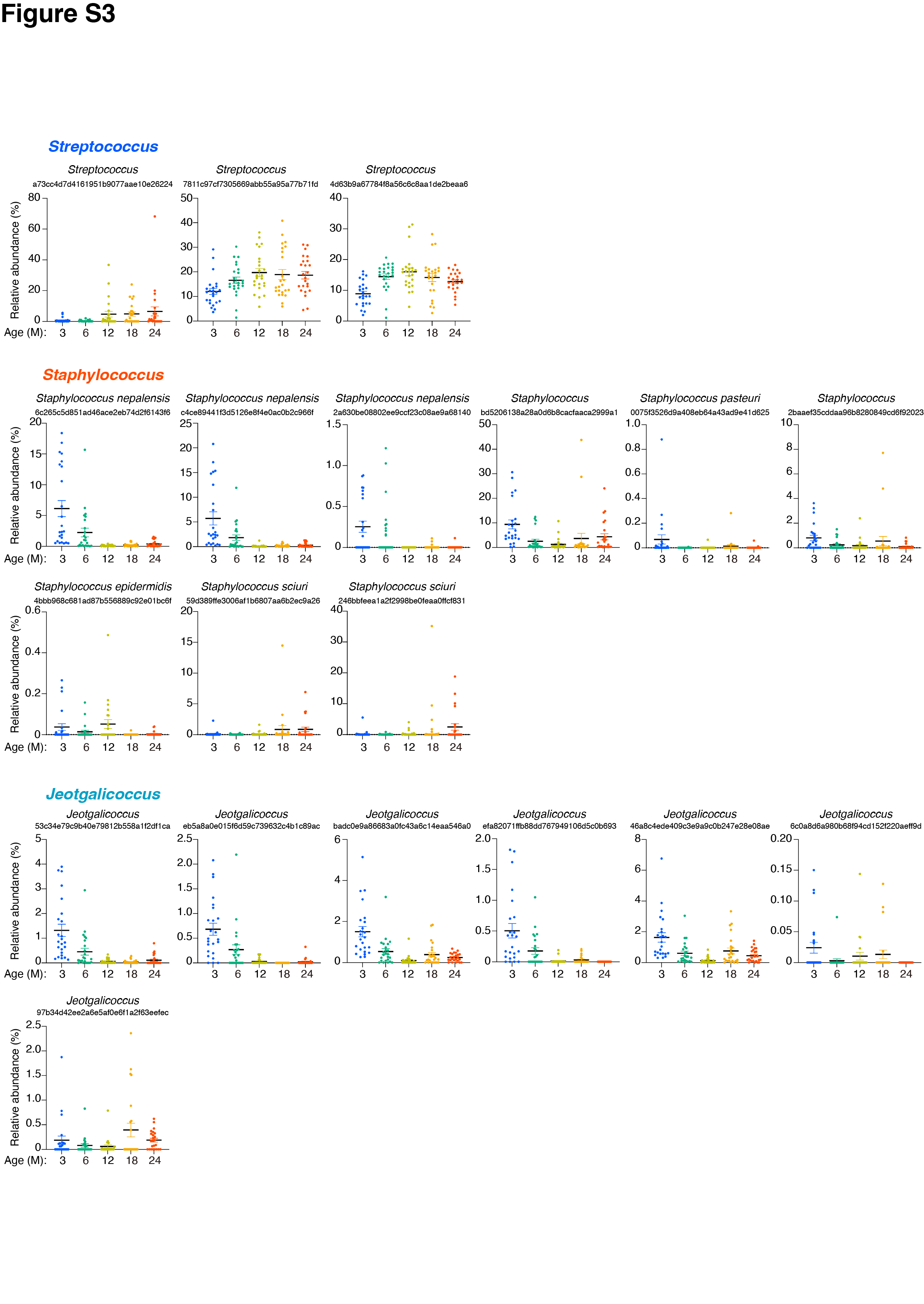


**Figure S3. Age-related changes in oral microbiota at species level**

In *Streptococcus*, *Staphylococcus*, and *Jeotgalicoccus*, Jitter plot shows the changes in the relative abundance of bacteria (ASVs) that were identified as statistically significant changes with aging by MaAsLin. The ASV number and name of the bacterial taxonomy classified by SILVA are represented on the top. Sample sizes (n) represent the number of biologically independent animals (*n* = 24). The data are indicated as means ± s.e.m.


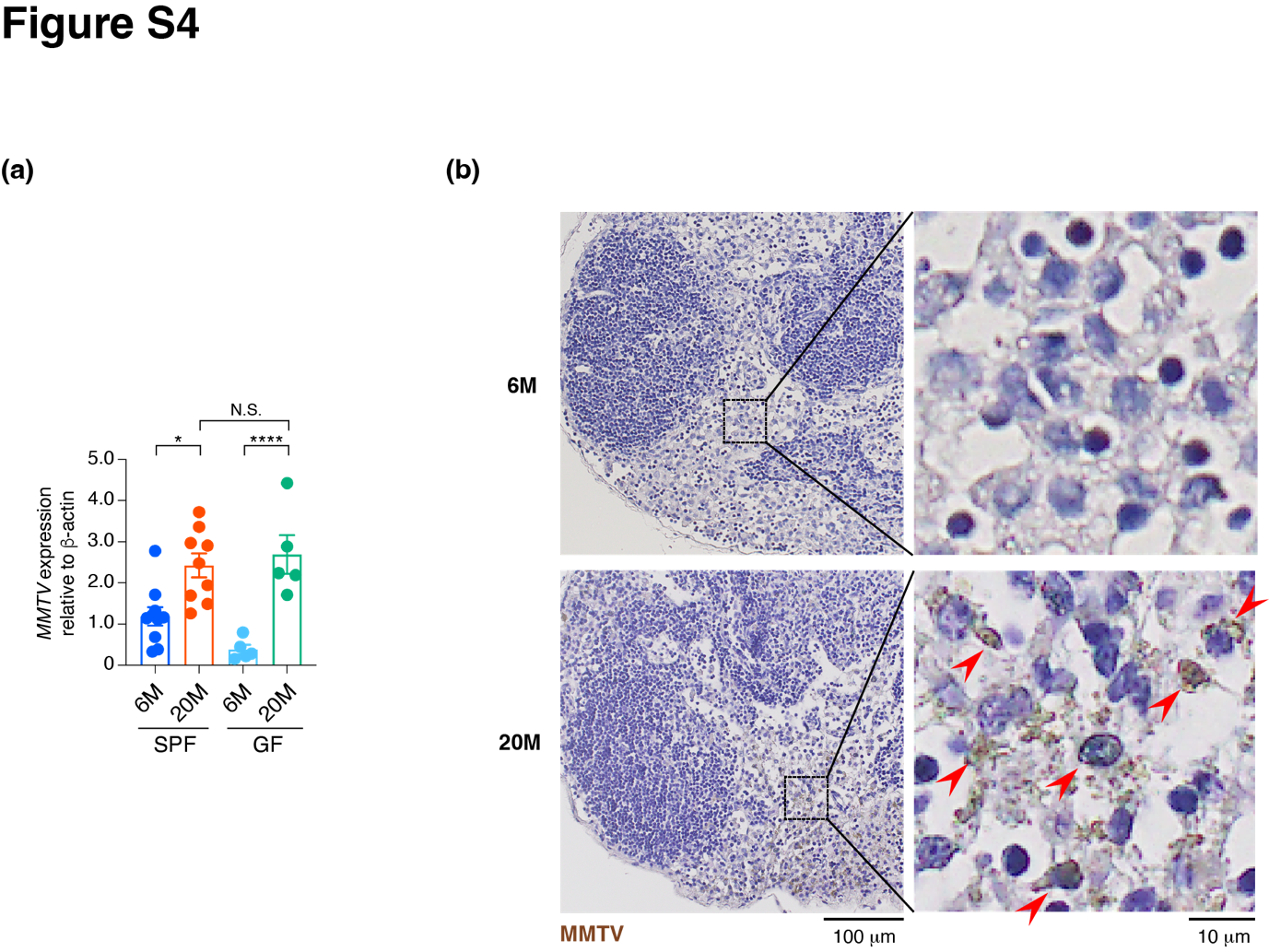


**Figure S4. Bacteria-independent *MMTV* expression in cervical lymph nodes with aging**

**(a)** The cervical lymph nodes isolated from SPF and GF mice (male) were subjected to analysis of RT-qPCR for *MMTV* expression. **(b)** Immunohistochemical images of cervical lymph nodes stained with MMTV from 6- or 20-month-old WT SPF mice (male). Scale bar, 100 μm. Sample sizes (n) indicate the number of biologically independent animals (*n* = 5 to 10 in **(a)**). The data are indicated as means ± s.e.m. Statistical significance was determined with two-way ANOVA followed by Šídák’s multiple comparisons test (**a**). M, months; N.S., not significant. * *p* < 0.05, ** *p* < 0.01, *** *p* < 0.005, **** *p* < 0.001.


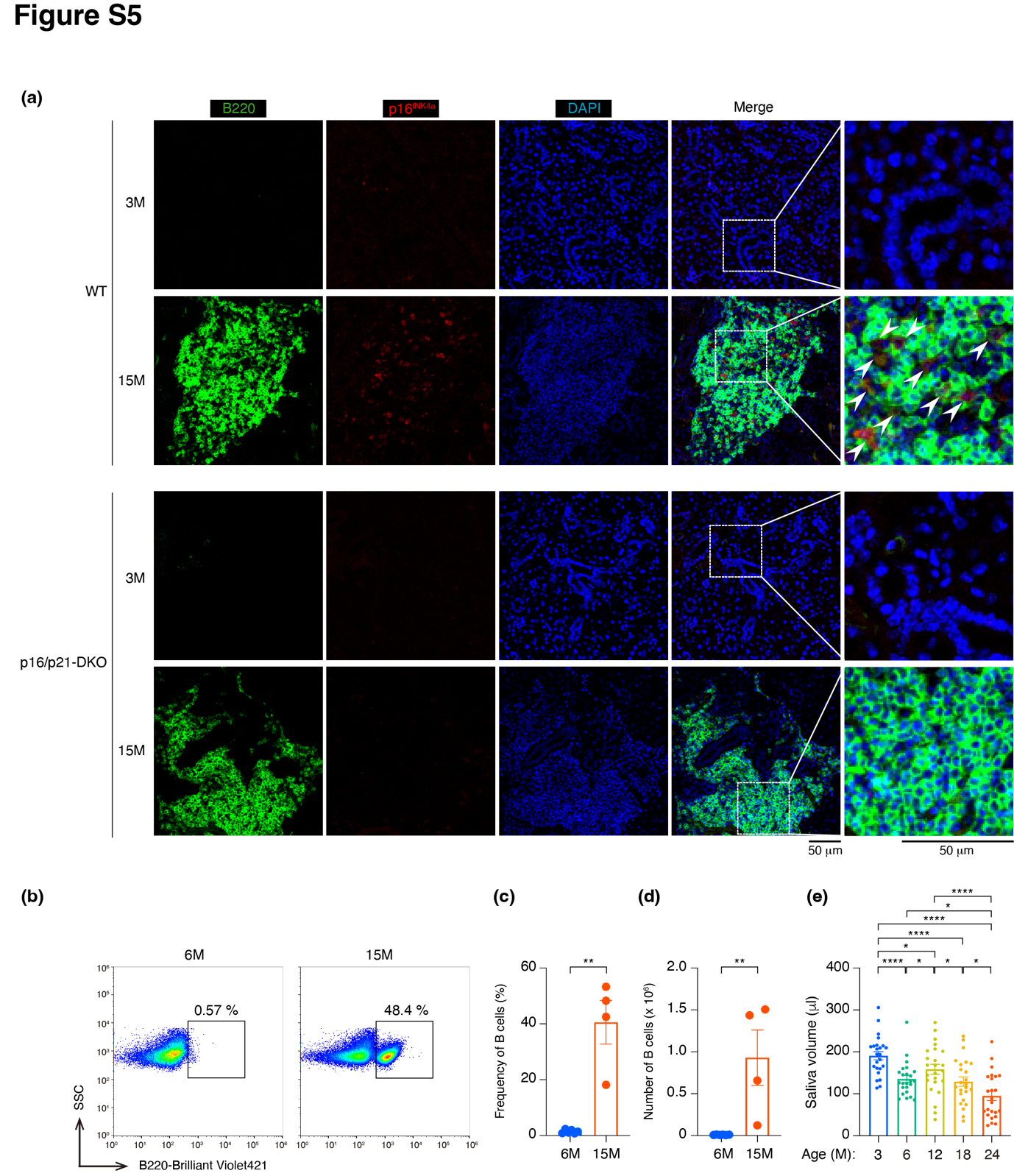


**Figure S5. Histological changes in** **submandibular salivary gland with aging**

**(a)** Immunohistochemical images of the submandibular salivary gland from 3- or 15-month-old WT and p16/p21-DKO mice (female) stained with antibodies against B220 and p16^INK4a^.

Scale bar, 50 μm. **(b-d)** Representative FACS profiles **(b)**, frequency **(c)**, and number **(d)** of B cells (B220^+^) in the submandibular salivary gland of 6- and 15-month-old SPF WT mice (female). **(e)** The volume of saliva collected at each month old (3, 6, 12, 18, and 24 months old) of the same individual WT mice (**Fig. 3**) was measured. Sample sizes (n) indicate the number of biologically independent animals (*n* = 4 or 6 in **(c, d)**, *n* = 24 in **(e)**). The data are indicated as means ± s.e.m. Statistical significance was determined with the Mann-Whitney *U* test **(c, d)** and one-way analysis of variance (ANOVA) followed by pairwise two-tailed paired *t* test **(e)**. M, months. * *p* < 0.05, ** *p* < 0.01, *** *p* < 0.005, **** *p* < 0.001.
